# Innovations in skeletal muscle regeneration: from physiology to bioengineering approaches for repair and restoration

**DOI:** 10.3389/fphys.2026.1789642

**Published:** 2026-04-17

**Authors:** Kamal Awad, Julia Aguirre, Misturat Adegbite, Akhilla Sajeev Kumar, Ahmed S. Yacoub, Mingxin Xia, Marco Brotto

**Affiliations:** 1 The Bone-Muscle Research Center, The College of Nursing and Health Innovations, The University of Texas at Arlington, Arlington, TX, United States; 2 Department of Bioengineering, College of Engineering, The University of Texas at Arlington, Arlington, TX, United States; 3 Department of Kinesiology, The College of Nursing and Health Innovations, The University of Texas at Arlington, Arlington, TX, United States; 4 Department of Graduate Nursing, The College of Nursing and Health Innovations, The University of Texas at Arlington, Arlington, TX, United States

**Keywords:** engineering innovation, hydrogels, muscle grafts, myogenesis, physiology, regeneration, skeletal muscle, volumetric muscle loss (VML)

## Abstract

Skeletal muscle is a dynamic tissue essential for voluntary movement, metabolism, and thermoregulation. Yet, its intrinsic regenerative capacity is overwhelmed in volumetric muscle loss (VML), where damage exceeds the native repair threshold. Conventional treatments such as muscle flaps and grafts provide only partial structural and functional recovery, underscoring the need for regenerative strategies that more precisely recapitulate the molecular and cellular physiology of muscle healing. This review first outlines the physiology of injury and muscle regeneration, with emphasis on key molecular pathways that govern inflammation, fibrosis, and myogenesis in VML. Building on this biological framework, we then examine hydrogels as soft material platforms for skeletal muscle tissue engineering, including: (i) acellular hydrogels and nanoparticle−loaded hydrogels designed to modulate the biochemical and biophysical microenvironment; (ii) cell−loaded hydrogels that deliver myogenic or stem/progenitor cell populations; and (iii) drug−loaded hydrogels for localized, sustained release of growth factors, cytokines, nucleic acids, or small molecules. Finally, we discuss emerging directions, including nanoparticle−integrated systems, dynamically stiffening or softening hydrogels, and advanced biofabrication approaches, and consider how these cellularized and acellular drug−, cell−, or nanoparticle−loaded hydrogels can be strategically leveraged to treat complex skeletal muscle injuries.

## 1 Introduction

Skeletal muscle is the predominant tissue in the human body, accounting for approximately 40-50% of total adult body mass, and it plays a critical role in locomotion, thermoregulation, and metabolic homeostasis. It also serves as a major reservoir for amino acids and energy substrates (Frontera and Ochala, 2015). The socioeconomic burden of musculoskeletal injuries is substantial, with treatment costs in the United States alone rising from an estimated $98 billion to $214 billion, with a rise of 117% between 1996 and 2014 (Peper et al., 2021), with the 2014 estimate corresponding to approximately 295 billion dollars in 2026 when adjusted for inflation.

Structurally, skeletal muscle is composed of post-mitotic, multinucleated muscle fibers formed through the fusion of mononucleated myoblasts. Following minor injury, skeletal muscle displays a robust regenerative capacity, primarily mediated by satellite cells (SCs), quiescent progenitors residing between the sarcolemma and basal lamina. Upon injury, SCs activate, proliferate, and differentiate into myoblasts that fuse to restore damaged fibers and contribute to muscle growth and homeostasis (Ma et al., 2024). SCs further regulate this process by coordinating inflammation, clearing necrotic debris, and releasing trophic signals that support the activation and function of mesenchymal stem cells.

Despite this regenerative efficiency, severe injuries such as volumetric muscle loss (VML), defined as the loss of ≥20% of muscle volume, overwhelm the intrinsic repair process. Skeletal muscle injuries are among the most common traumas, frequently resulting from high-impact traffic accidents, acute or chronic peripheral arterial occlusion, surgical interventions, or sport-related contusions (Souza and Gottfried, 2013; Ma et al., 2024). VML is one of the most debilitating musculoskeletal injuries in the military, as 92% were identified in a cohort of battlefield-injured military personnel (Garg et al., 2015). Similarly, chronic muscle degeneration due to genetic disorders (muscular dystrophies) and age-related sarcopenia leads to incomplete or delayed healing. These conditions are characterized by a depletion of SCs and disruption of the ECM, which together impair innate regeneration. As a result, the tissue becomes prone to chronic inflammation, fibrosis, and deterioration.

Current clinical strategies are extremely limited (Gilbert‐Honick and Grayson, 2020), such as autologous muscle flaps (Vekris et al., 2008), offering limited success in fully restoring muscle function (Tu et al., 2008; Lee and Mun, 2014). This underscores the urgent need for bioengineered solutions that can restore both muscle tissue structure and function. Skeletal muscle tissue engineering (SMTE) has emerged as an innovative approach, enabling the development of bio-artificial constructs that can support, enhance, and potentially accelerate the regeneration process (Liu et al., 2018).

Among the various biomaterials explored within SMTE, hydrogels have garnered significant attention. Their minimally invasive delivery, tunable biochemical and biomechanical properties, and ability to encapsulate and deliver therapeutic cells and growth factors make them capable of addressing the multifactorial challenges of muscle regeneration. By mimicking key biochemical and biophysical features of the ECM, including integrin−binding motifs and tissue−like viscoelasticity, hydrogels promote cell adhesion, proliferation, and lineage−specific differentiation, including myogenesis (Tibbitt and Anseth, 2009; Caliari and Burdick, 2016; Morwood et al., 2023). Continued innovation in hydrogel design and bio-fabrication will be essential to achieving clinically translatable therapies that restore and replicate native skeletal muscle structure and function.

## 2 From injury to renewal: the sequential phases of skeletal muscle regeneration

Skeletal muscle (SKM) possesses a remarkable capacity to regenerate following injury, largely due to its resident stem cells known as satellite cells (also referred to as muscle stem cells, or MuSCs) (Relaix and Zammit, 2012; Relaix et al., 2021). Under normal conditions, SCs remain quiescent beneath the basal lamina of muscle fibers, characterized by expression of the marker Paired box protein 7 (Pax7). Upon injury, they become activated to repair damaged fibers (Relaix et al., 2021). The regeneration process proceeds through several coordinated and overlapping phases involving inflammation, activation, differentiation, and remodeling.

**Figure 1:**
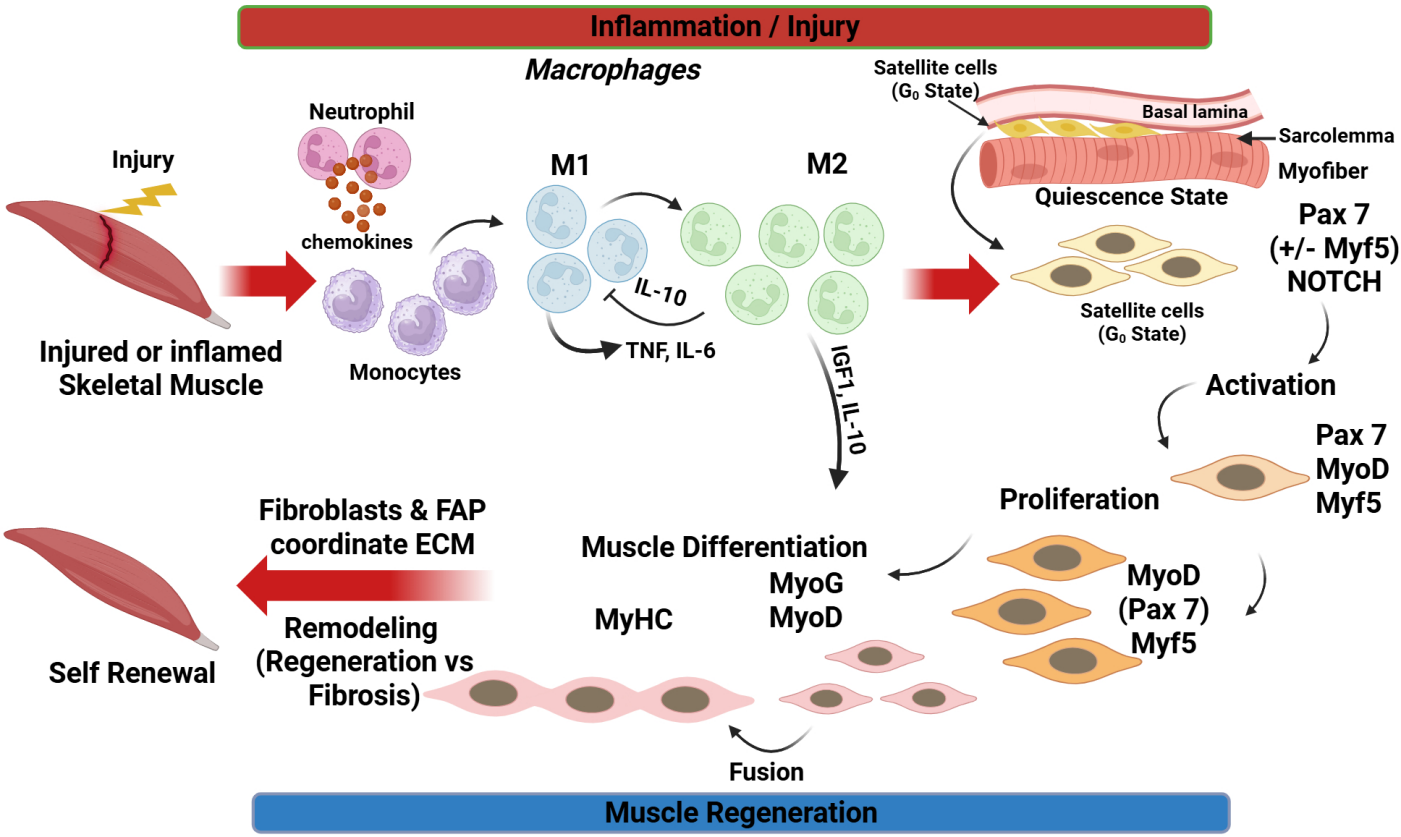
Inflammation‐driven regulation of skeletal muscle regeneration. Following injury, neutrophils and classically activated M1 macrophages infiltrate the damaged tissue, clearing debris and secreting pro-inflammatory cytokines such as TNF and IL-6. Subsequent polarization toward M2 macrophages and the release of IL-10 and IGF-1 promote inflammation resolution and support myogenic differentiation. Muscle stem cells (satellite cells) transition from a quiescent Pax7^+^/Notch-dependent state to activated, proliferative progenitors expressing MyoD and Myf5, then differentiate (MyoD^+^/MyoG^+^, MyHC^+^) and fuse to form new myofibers. At the same time, a subset self-renews to replenish the stem cell pool. Fibroblasts and fibro-adipogenic progenitors (FAPs) coordinate extracellular matrix (ECM) remodeling, providing a transient stromal niche that can either facilitate effective muscle regeneration or, if dysregulated, contribute to fibrosis. Created in https://BioRender.com.

Muscle damage, such as that caused by volumetric muscle loss (VML), leads to myofiber necrosis and initiates an acute inflammatory response. Within hours (approximately 1–3 hours post-injury), neutrophils infiltrate the injured site, clear necrotic debris, and secrete chemokines that recruit circulating monocytes, which subsequently differentiate into macrophages (Teixeira et al., 2003; Tidball, 2017). During the first days after injury, pro-inflammatory M1 macrophages clear necrotic material and activate SCs (Brigitte et al., 2010; Koike et al., 2022; Zhu et al., 2023b). Then gradually transition to reparative M2 phenotypes that secrete trophic factors (e.g., IGF-1, IL-10) to support tissue repair (Hoffman et al., 2022; Qi et al., 2025). This timely shift from M1 to M2 is essential, as macrophage depletion impairs regeneration, whereas M2 macrophages facilitate the repair phase (**Figure 1**).

Following the initial inflammatory response, signals from growth factors such as hepatocyte growth factor (HGF) and fibroblast growth factor-2 (FGF-2), and cytokines released from immune cells, stimulate quiescent Pax7^+^ SCs to exit their G_0_ state. Within 24 hours, many become activated and co-express Pax7 with myogenic regulatory factors (MRFs) such as Myogenic Differentiation (MyoD) and Myogenic Factor 5 (Myf5) (Relaix and Zammit, 2012; Koike et al., 2022). Under physiological conditions, Notch signaling maintains SC quiescence, but upon injury, Notch activity decreases while activating pathways that elevate MyoD and Myf5 expression, committing the cells toward the myogenic lineage (Relaix and Zammit, 2012). Over the following days, proliferating myoblasts begin to exit the cell cycle and differentiate into mature muscle cells (**Figure 1**). This stage is characterized by downregulation of Pax7 and induction of differentiation markers such as Myogenin (MyoG) and late-stage MRFs, including MRF4 (Koike et al., 2022). Myoblasts align and fuse to form multinucleated myotubes or with existing muscle fibers, a process enhanced by cytokines such as IL-4 and IL-13 (Qi et al., 2025). By approximately 4–7 days post-injury, nascent myotubes expressing embryonic myosin heavy chain (MyHC) are visible and begin to exhibit contractile properties. Anti-inflammatory M2 macrophages and fibro/adipogenic progenitors (FAPs) further support this stage by secreting pro-myogenic growth factors, including IGF-1, IGF-2, and TGF-β3, while reorganizing the extracellular matrix (ECM) to facilitate fiber formation. A subset of proliferating SCs retains Pax7 expression and re-enters quiescence, replenishing the stem cell reservoir for future repair (Hoffman et al., 2022).

During the final maturation and remodeling phase, which occurs over one to three weeks, newly formed myotubes mature into functional muscle fibers, and additional myoblasts fuse with both nascent myotubes and surviving injured fibers, contributing to fiber growth and the restoration of contractile capacity. Fibroblasts and FAPs coordinate ECM remodeling by depositing and refining collagenous matrix, reconstructing the basal lamina, and restoring proper muscle architecture (Morgan and Partridge, 2003; Zammit et al., 2004; Mann et al., 2011; Lemos et al., 2015). Concurrently, revascularization ensures sufficient blood supply to the regenerating tissue, while reinnervation re-establishes neuromuscular junctions between new fibers and motor neurons (Lee et al., 2000; Fukada et al., 2007; Kuang and Rudnicki, 2008; Gnocchi et al., 2009). The connective tissue sheath and tendon attachments are also rebuilt during this stage (Kääriäinen et al., 2000; Morgan and Partridge, 2003; Zammit et al., 2004; Mann et al., 2011; Lemos et al., 2015). By the end of the remodeling phase, muscle structure and function are largely restored, highlighting the remarkable regenerative capacity of skeletal muscle tissue.

## 3 Molecular control of muscle repair and regeneration

The process of skeletal muscle regeneration is orchestrated by a network of genes, signaling pathways, and cytokines that dynamically regulate satellite cell activation, proliferation, differentiation, and self-renewal as presented in **Figure 1**. Among these, the transcription factor *Pax7* serves as the canonical marker of satellite cells (Relaix et al., 2006; Der Vartanian et al., 2019). *Pax7* maintains the stem cell pool and is indispensable for muscle regeneration; mice lacking *Pax7* fail to preserve a functional satellite cell population into adulthood, resulting in regenerative failure. Upon activation, Pax7^+^ satellite cells upregulate myogenic regulatory factors (MRFs), a family of muscle-specific transcription factors that orchestrate myogenesis and repair. Key MRFs include Myf5 and MyoD, which mark the early differentiation phase, Myogenin, essential for terminal differentiation, and MRF4, which contributes to myofiber maturation (Relaix et al., 2006; Buckingham and Relaix, 2015; Der Vartanian et al., 2019; Relaix et al., 2021). Myf5 and MyoD are often co-expressed in early-activated satellite cells, excessively committing them to the myogenic lineage. MyoD, often termed the “master regulator” of myogenesis, activates muscle-specific genes and drives re-entry into the cell cycle. As myoblasts exit proliferation, MyoG expression increases, promoting the synthesis of structural proteins and fusion of myoblasts into multinucleated myotubes. Cells committed to differentiation downregulate *Pax7* and permanently exit the stem state (George-Weinstein et al., 1993; Schultz and McCormick, 1994; Cornelison and Wold, 1997; Creuzet et al., 1998; Hernández-Hernández et al., 2017). Mature myotubes and newly formed fibers then express genes encoding contractile proteins, such as MyHC and actin, as well as developmental isoforms, such as MYH3, as the muscle fibers mature.

### 3.1 Notch signaling

In uninjured muscle, Notch signaling preserves satellite cell quiescence. Delta-like ligands from neighboring muscle fibers or endothelial cells activate canonical Notch pathways in SCs, maintaining *Pax7* expression and preventing premature differentiation (Bjornson et al., 2012; Mourikis et al., 2012; Wang and Rudnicki, 2012; Motohashi and Asakura, 2014; Quarta et al., 2016; Tierney and Sacco, 2016; Low et al., 2018; Verma et al., 2018; Relaix et al., 2021). Disruption of Notch signaling causes spontaneous differentiation and depletion of the stem cell pool. Following injury, Notch activity declines while pro-differentiation signals, such as Wnt, increase, thereby allowing SC activation. Notch also regulates the balance between self-renewal and differentiation through downstream targets, including KLF7 and miR-708, which modulate quiescence and cell cycling (Relaix et al., 2021). Proper regulation of Notch signaling ensures that a subset of activated SCs reverts to quiescence (re-expressing *Pax7*), preserving long-term regenerative capacity.

### 3.2 Inflammatory cytokines

Inflammatory cytokines released during the early post-injury phase play a dual role in myogenesis. Tumor necrosis factor-alpha (TNF-α) and interleukin-6 (IL-6), secreted by infiltrating M1 macrophages (and to some extent by damaged muscle fibers), stimulate satellite cells at controlled levels. These cytokines encourage myoblast proliferation and early differentiation, in part by inducing the expression of MyoD and other factors (Dhawan and Rando, 2005; Grefte et al., 2007). For example, IL-6 acts on muscle cells to promote growth and repair. At the same time, TNF-α facilitates clearance of inhibitory stromal cells, such as fibro/adipogenic progenitors (FAPs), by inducing apoptosis, thereby creating a pro-regenerative microenvironment (Li et al., 2004; Lemos et al., 2015). However, chronic elevation of these cytokines can be harmful, causing tissue damage and stem cell exhaustion. Thus, the timely shift to an anti-inflammatory, pro-regenerative environment is important for successful repair (**Figure 1**).

### 3.3 Growth factors and regenerative cues

Following injury, numerous growth factors are released from the ECM, injured fibers, and infiltrating immune cells. Hepatocyte growth factor (HGF) is one of the earliest signals to activate quiescent SCs. Basic fibroblast growth factor (FGF-2) acts as a mitogen, promoting SC proliferation. Insulin-like growth factor-1 (IGF-1), secreted during later stages, enhances myoblast differentiation, survival, and hypertrophy. Additional factors, such as platelet-derived growth factor (PDGF) and leukemia inhibitory factor (LIF), further support myoblast survival and expansion (Relaix and Zammit, 2012). Collectively, multiple studies have identified IGF-1, FGF, HGF, PDGF, LIF, IL-6, and TNF-α among the factors that promote satellite cell activation, proliferation, and differentiation during regeneration (Schultz and McCormick, 1994; Li et al., 2004; Fu et al., 2015; Hernández-Hernández et al., 2017). In contrast, transforming growth factor-beta 1 (TGF-β1) functions as an inhibitory factor in muscle repair by promoting fibroblast activation and scar formation at the expense of muscle fiber regeneration. High TGF-β levels, often from M2 macrophages and FAPs during late regeneration, suppress myoblast proliferation and differentiation (Yin et al., 2013; Fu et al., 2015). Therefore, maintaining a delicate balance between pro-myogenic (e.g., IGF-1, HGF, FGF) and pro-fibrotic (e.g., TGF-β1) signals is essential to prevent scar formation and ensure proper tissue regeneration.

### 3.4 Cell–cell interactions within the regenerative niche

Muscle regeneration is a multicellular process reliant on the coordinated actions of diverse cell types. In addition to immune cells, vascular endothelial cells support muscle repair by releasing trophic factors such as VEGF, promoting angiogenesis and SC activation, and expressing ligands that influence satellite cell fate (George-Weinstein et al., 1993; Schultz and McCormick, 1994; Cornelison and Wold, 1997; Creuzet et al., 1998; Wang and Rudnicki, 2012; Fu et al., 2015; Webster et al., 2016). Likewise, fibroblasts and FAPs provide the structural ECM scaffold for regenerating fibers and secrete Wnt proteins and other pro-myogenic cues that promote differentiation once sufficient myoblasts accumulate. However, dysregulated activation of these fibrogenic cells leads to excessive collagen deposition and scar tissue formation. Other supporting players include mast cells and eosinophils, which arrive early and secrete IL-4, which signals the activation of FAPs. In contrast, Schwann cells and other glial cells aid in reinnervation during later stages of remodeling. Together, these complex cell–cell interactions ensure temporal coordination: inflammatory signals initiate SCs activation, pro-differentiation factors (e.g., IL-4, IGF-1) drive fusion, and remodeling enzymes such as matrix metalloproteinases (MMPs) ensure structural restoration of the muscle (Kääriäinen et al., 2000; Zammit et al., 2004; Mann et al., 2011; Motohashi and Asakura, 2014; Lemos et al., 2015).

## 4 Impaired regeneration after volumetric muscle loss

As stated above, VML refers to the traumatic loss of a substantial portion of skeletal muscle, including nerve and vascular, typically exceeding 20% of muscle mass, such that the native regenerative capacity is overwhelmed (Corona et al., 2016). These large-scale injuries often result from battlefield trauma, open fractures, or severe surgical resections. Whereas minor muscle wounds can regenerate through coordinated activation of satellite cells and remodeling of the ECM, VML injuries heal aberrantly. Instead of functional muscle restoration, VML typically leads to chronic deficits characterized by extensive fibrosis, limited muscle fiber regeneration, and poor functional recovery (Hoffman et al., 2022; Zhu et al., 2023b). The defect region becomes filled with non-contractile connective tissue rather than new myofibers, rendering it essentially incapable of endogenous repair.

### 4.1 Loss of the regenerative niche

A defining feature of VML is the complete loss of structural and cellular elements critical for regeneration. The injury often removes entire sections of muscle tissue along with their basal lamina, vasculature, and innervation. This destruction eliminates resident satellite cell populations and the native ECM framework that supports their activation and differentiation (Grogan and Hsu, 2011; Tidball, 2011; Garg et al., 2015; Grasman et al., 2015b; Corona et al., 2018; Forcina et al., 2019). Without this regenerative niche, myogenic progenitors lack a scaffold for adhesion and mechanical cues to guide alignment and growth. The remaining satellite cells at the periphery may attempt regeneration, but they are often unable to bridge the large defect or sustain proliferation across the fibrotic gap. Consequently, organized muscle regeneration fails to occur, leaving behind an acellular void that favors stromal cell infiltration and fibrosis.

### 4.2 Aberrant inflammation and fibrosis

As a result, VML injuries provoke a prolonged and dysregulated inflammatory response that skews healing toward fibrosis rather than regeneration. The balance of pro-regenerative and fibrogenic signaling becomes disrupted. Regenerative cytokines such as IGF-1, HGF, and FGF-2, essential activators of SCs’ proliferation, are frequently downregulated or absent within the VML wound milieu (Allen et al., 1995; Chargé and Rudnicki, 2004; Wozniak and Anderson, 2007; Nuutila et al., 2017; Forcina et al., 2019). Similarly, moderate levels of TNF-α and IL-6, which can promote myogenesis under controlled conditions, become deficient or ineffective in large defects. In contrast, TGF-β1 expression is markedly elevated following VML. Excessive TGF-β1 signaling drives fibroblast activation and extracellular matrix deposition, fostering scar formation rather than muscle regeneration. Disruption of macrophage polarization and impaired TNF-α–mediated apoptosis of fibro-adipogenic progenitors (FAPs) further perpetuates fibrosis. As a result, collagen accumulation stiffens the tissue, restricts vascular infiltration, and inhibits nascent myofiber extension. Once this fibrotic cascade dominates, the microenvironment becomes irreversibly hostile to myogenesis.

### 4.3 Lasting functional deficits

The clinical consequence of VML goes beyond morphological scarring. The combination of myofiber loss, excessive fibrosis, and lack of vascularization and innervation translates to substantial and permanent functional impairment. Patients experience reduced contractility, diminished range of motion, and, in severe cases, the development of muscle contractures. Atrophy of the surrounding intact muscle fibers often accompanies these deficits due to disuse and disrupted mechanical loading (Hoffman et al., 2022). Unlike minor lacerations that eventually regain function, VML represents a threshold beyond which intrinsic muscle regeneration fails, leading to chronic disability even with extensive rehabilitation efforts.

### 4.4 Therapeutic strategies and emerging interventions

Given the intrinsic limitations of natural repair, VML requires medical intervention rather than relying on natural healing. Conventional approaches, such as autologous muscle transfers, can partially restore volume and function but suffer from donor site morbidity and limited integration (Zhu et al., 2023b). Recently, regenerative medicine strategies have shown promise in addressing the challenges of VML. These approaches seek to recreate the regenerative niche by combining cellular, biochemical, and structural cues to promote myogenesis while suppressing fibrosis.

Tissue-engineered scaffolds and biomaterial constructs are being designed to recruit progenitor cells and modulate the immune microenvironment, with some success in animal models (Tidball, 2011; Garg et al., 2015; Nuutila et al., 2017; Forcina et al., 2019). Incorporation of growth factors or stem cells aims to reintroduce pro-myogenic signals and structural guidance. Pharmacological efforts target the fibrotic response, with interventions that inhibit TGF-β signaling and complement rehabilitative exercise showing encouraging results in preclinical models (Li et al., 2004; Lemos et al., 2015; Whitaker et al., 2025). Ultimately, these combined strategies strive to shift the wound environment from a fibrotic to a regenerative state, restoring muscle architecture, vascularity, innervation, and function that are otherwise lost during natural healing (Greising et al., 2017; Corona et al., 2018).

## 5 Hydrogels as platforms for skeletal muscle regeneration

Hydrogels are hydrophilic, three-dimensional polymeric networks that provide structural support while mimicking the biochemical cues of native ECM to promote myogenesis and functional skeletal muscle regeneration. Injectable and self-healing hydrogels enable homogeneous loading, conform to irregular defect shapes, and minimize patient discomfort (Lee and Mun, 2014). Furthermore, hydrogels are amenable to processing into different design configurations using advanced microfabrication techniques such as freeze drying, electrospinning, 3D printing, molding, and direct injection. The ideal hydrogel for SMTE should be biocompatible, biodegradable, and possess chemical and mechanical properties that support cellular growth and fusion to form parallel, aligned muscle fibers that replicate the architecture and contractile strength of native skeletal muscle. Its elastic modulus can be tuned via chemical, thermal, or photo-crosslinking methods to match that of native skeletal muscle (~10–12 kPa), thereby promoting myofiber maturation (Camci-Unal et al., 2014; Leijten et al., 2017; Guo et al., 2019; Xie et al., 2020; Fischer et al., 2021). Hydrogel microfibers can be used both as individual self-supporting structures and as building-block units to assemble full-scale three-dimensional skeletal muscle constructs (Khademhosseini and Langer, 2007). Additionally, design elements such as scaffold porosity and dynamic mechanical stimuli have been shown to promote myofiber alignment and maturation, which are critical for restoring contractile function. Recent studies have demonstrated that cellularized hydrogels incorporating myoblasts, mesenchymal stem cells (MSCs), or endothelial cells improve myogenic differentiation, vascularization, and integration with host tissues in preclinical models. These constructs also enable the co-delivery of bioactive agents, such as growth factors, cytokines, and extracellular vesicles, which help shape the regenerative microenvironment. Given these multifaceted capabilities, hydrogels are considered the first-choice biomaterials for SMTE (Drury and Mooney, 2003). As the field advances, the continued refinement of hydrogel design and fabrication strategies will be vital for developing clinically translatable therapies that restore the structure and function of damaged skeletal muscle. Despite this promise, most hydrogel systems have yet to demonstrate scalable manufacturing, robust vascular and neural integration, or durable contractile restoration in large−animal VML models, underscoring the gap between preclinical innovation and clinical translation. In this section, hydrogels are broadly classified into natural, synthetic, and hybrid (composite) systems, based on their origin and cross-linking mechanisms.

### 5.1 Natural hydrogels

Natural hydrogels, sourced from biological polymers such as collagen, fibrin, gelatin, Alginate, hyaluronic acid, and decellularized extracellular matrix (dECM), have become foundational in SMTE due to their biocompatibility, biodegradability, and structural similarity to native tissues. These biomaterials typically elicit minimal inflammatory responses and actively promote muscle regeneration (Drury and Mooney, 2003; Onoe et al., 2013; Pollot et al., 2018). In particular, hydrogels derived from the ECM are enriched with a variety of bioactive motifs, notably arginine-glycine-aspartic acid (RGD), which promote cell adhesion and growth (Baker et al., 2017; Bootsma et al., 2017).

Collagen, the predominant fibrous protein and primary component of the ECM, accounts for 25−35% of protein content in the body (De France et al., 2017). Collagen exhibits desirable properties such as good biodegradability and biocompatibility, support for cell adhesion, promotion of tissue healing, and induction of cell polarization (Costantini et al., 2017; Villa et al., 2017). Studies have shown that collagen scaffolds can facilitate myoblast infiltration and differentiation into muscle fibers, as well as the revascularization of damaged muscle tissues (Jo et al., 2017; Fischer et al., 2021). As the most used form in SMTE, Type I collagen can be extracted from various biological tissues, such as skin, ligament, cartilage, and bone, using enzymatic or acid/base processing methods. Type I collagen hydrogels closely mimic the native environment of skeletal muscle tissue. However, their relatively high stiffness hinders long-term muscle culture, differentiation, and contractility (Pollot et al., 2018). These hydrogels are formed at physiological temperature (37 °C), which induces the self-assembly of solubilized type I collagen fibrils (De France et al., 2017). Although they exhibit good elasticity, collagen hydrogels degrade rapidly if not stabilized. To address this limitation, cross-linking agents are used to enhance mechanical stability.

Gelatin is a denatured, low-cost derivative of collagen produced through partial hydrolysis and thermal denaturation. As a collagen derivative, it retains essential cell-binding motifs, enabling effective cell adhesion and proliferation. Additionally, gelatin exhibits lower immunogenicity than native collagen because it lacks aromatic amino acid residues removed during denaturation. This makes it particularly attractive for tissue engineering applications. Gelatin hydrogels can be crosslinked using microbial transglutaminase (MTG), an FDA-approved enzyme, which has been shown to improve the long-term culture and maturation of myotubes (Boso et al., 2020). Gelatin exhibits thermoresponsive behavior, forming physically cross-linked hydrogels below 30−35 °C and dissolving into a single coil at physiological temperature. To improve mechanical strength and stability, cross-linking agents such as transglutaminase or genipin are often used. Gelatin’s chemical versatility allows it to be combined with synthetic polymers, nanoparticles, and growth factors to enhance electrical conductivity, bioactivity, and vascularization. Despite these advantages, gelatin does present challenges. As a naturally derived product, gelatin can vary between batches, compromising reproducibility. Additionally, the degradation rate is rapid *in vivo*, unless cross linking is optimized (Catoira et al., 2019). In current research, gelatin is functionalized with methacrylate groups to form a photosensitive methacrylate gelatin (GelMA), a photosensitive material that enhances the structural integrity and mechanical properties of the scaffold (Fischetti et al., 2020).

Fibrin is another widely used natural material in SMTE valued for its rich cell-binding motifs, enzymatic degradability, and ability to sequester and present growth factors (Fischetti et al., 2020). Fibrin hydrogels have intrinsic angiogenic properties, which promotes the vascularization of skeletal muscle constructs upon *in vivo* implantation. They possess specific binding sites for angiogenic growth factors, such as basic fibroblast growth factor−2 (bFGF−2) and insulin−like growth factor−1 (IGF−1), both of which are known to promote myogenesis. Fibrin also acts as a natural, temporary matrix during wound healing, enhancing tissue repair and regeneration (Pollot et al., 2018). In SMTE applications, fibrin hydrogels have been shown to support co-culture of muscle and endothelial cells, enabling the formation of aligned myofibers and interconnected endothelial networks (Lin et al., 2019). For example, Marcinczyk et al. demonstrated that fibrin hydrogels incorporating laminin-111 promoted myoblast proliferation, increased secretion of regenerative growth factors, and responded positively to electromechanical stimulation, thereby further enhancing IGF-1 and VEGF expression (Marcinczyk et al., 2017). Similarly, Gilbert Honick and colleagues evaluated myoblast-seeded electrospun fibrin scaffolds in a murine VML injury model with defects to the tibialis anterior muscle (Gilbert-Honick et al., 2018; Kim et al., 2019). Subsequent studies also reported that fibrin microfilament scaffolds support myofibril growth and improve the regeneration of large-scale skeletal muscle defects *in vivo (*Kozan et al., 2023; Li et al., 2023).

Natural polysaccharide-based hydrogels are being investigated for skeletal muscle repair and regeneration due to their biocompatibility and minimal immunogenicity. Natural materials from mammals, such as collagen, fibrin, and gelatin, are suitable for SMTE due to their higher cell adhesion density, greater cell proliferation rate, retention of endogenous growth factors, and biological signals for myogenic differentiation (Guo et al., 2024). Despite these advantages, natural hydrogels fall short in replicating the biochemical, structural, and mechanical complexity of ECM. For example, alginate, a natural polysaccharide known for its biocompatibility, biodegradability, and non-immunogenicity, lacks inherent cell adhesion. As a result, poor tissue interactions and insufficient regenerative behavior may occur with alginate-based hydrogels (Broer et al., 2020). Decellularized extracellular matrices (dECMs) hydrogels have also shown promise in VML repair. In preclinical models and early human trials, dECM materials have supported *de novo* muscle fiber formation and functional recovery. However, some reports suggest that improvements in force production may be due to enhanced force transmission through fibrotic scar tissue rather than to muscle regeneration (Gupta et al., 2021). Other reports highlight the role of dECM in indirectly promoting a pro-regenerative environment through local immune modulation (Samandari et al., 2022). Inherent drawbacks of dECM materials implants include batch-to-batch variability, lack of control over biophysical and biochemical properties, and nanostructural features such as porosity. These hydrogels are typically cross-linked and molded into homogenous gel constructs, which are implanted at the VML defect site using sutures or adhesive agents to ensure their stable retention. While natural polymer-based hydrogels are generally favored for their ability to support a cell-compatible microenvironment, they offer limited control over key properties, including processability, biofunctionality, and mechanical strength. Compared to synthetic hydrogels, natural polymers exhibit superior biocompatibility, biodegradability, intrinsic biological activity, and minimal cytotoxicity. However, their weak mechanical strength hinders their clinical translation. To overcome these limitations, natural hydrogels are blended with synthetic polymers or ceramics, or subjected to cross-linking reactions, to enhance mechanical properties and prolong degradation time.

### 5.2 Synthetic hydrogels

Synthetic hydrogels, engineered from non-biological polymers such as polyethylene glycol (PEG), polyacrylamide (PAAm), poly-ϵ-caprolactone (PCL), polylactic-co-glycolic acid (PLGA), and polydimethylsiloxane (PDMS), have been explored as alternatives to natural hydrogels in SMTE, though they remain less commonly used. These materials are typically formed through covalent or ionic cross-linking of synthetic monomers (e.g., PEG, polyacrylic acid), producing hydrophilic, three-dimensional polymer networks with tunable physical and chemical properties (Kim et al., 2022). One advantage of synthetic hydrogels is their customizable properties. Parameters such as stiffness, porosity, viscoelasticity, and degradation kinetics can be precisely adjusted by altering the polymer’s molecular weight, concentration, and crosslinking density (Page et al., 2011; Grasman et al., 2015a; Lev and Seliktar, 2018). Furthermore, their reproducibility, low immunogenicity, and resistance to batch-to-batch variability or pathogen transmission make them attractive for clinical translation, as they match the requirements of muscle tissue environments (Page et al., 2011; Lev and Seliktar, 2018). Despite lacking inherent bioactivity, synthetic hydrogels can be functionalized with bioactive motifs, such as short peptides, to promote cell adhesion and tissue integration. Their hydrophilic surfaces and chain mobility typically result in low protein adsorption, but this limitation can be overcome through covalent modification (Page et al., 2011; Grasman et al., 2015a). Synthetic hydrogels can be engineered to enable the controlled release of therapeutic molecules such as growth factors, thereby promoting muscle regeneration *in vivo*. However, challenges remain, as synthetic hydrogels exhibit weaker cell adhesion than their natural counterparts, and their degradation byproducts may trigger foreign-body responses. Moreover, fewer synthetic polymers have been studied extensively for SMTE applications than natural polymers. Among the most researched are PEG and PAAm, while others, such as PLGA, PCL, and PDMS, have shown potential but require further investigation (Grasman et al., 2015a; Heher et al., 2015; Catoira et al., 2019).

### 5.3 Hybrid (composite) hydrogels

Hybrid or composite hydrogels represent an innovative convergence of natural and synthetic materials, designed to harness the best attributes of both. By combining the mechanical robustness of synthetic polymers and the bioactivity of natural polymers, hybrid hydrogels offer a versatile platform for skeletal muscle regeneration. This approach enables the material to exhibit improved mechanical strength, biological activity, and functional versatility compared to conventional hydrogels (Gholobova et al., 2020). Enhanced mechanical properties, such as superior toughness, elasticity, and durability, make these hydrogels well-suited for biomedical applications requiring mechanical stability (Gholobova et al., 2020). Incorporating bioactive molecules or natural polymers into hybrid hydrogels enhances their ability to replicate the ECM, promoting cell adhesion, proliferation, and differentiation. Hybrid hydrogels also offer precise control over drug release kinetics, making them ideal for sustained or targeted therapeutic delivery. Many are engineered to be stimuli-responsive, enabling them to change properties in response to environmental factors like pH, temperature, or ionic strength, which is especially valuable in tissue engineering and drug delivery applications (Marcinczyk et al., 2017).

Hybrid (composite) hydrogels can be categorized based on the architecture of their polymer networks, including co-networks, interpenetrating networks (IPNs), and semi-interpenetrating networks (semi-IPNs). Co-networks are formed by copolymerizing methacrylate-functionalized synthetic polymers with natural polymers such as gelatin methacrylate (GelMA), often using photoinitiated radical polymerization. IPNs involve two or more polymer networks that are interlaced but not covalently bonded, while semi-IPNs consist of one crosslinked network interpenetrated by another linear or branched polymer (Gilbert-Honick et al., 2018).

Hybrid hydrogels can be classified into several types: reversible physical hydrogels formed through non-covalent interactions, chemically crosslinked hydrogels with covalent bonds linking different polymers, and nanocomposite hydrogels that integrate nanoparticles to improve mechanical strength or functional properties. Hybrid hydrogels are typically prepared using click chemistry, self-assembly, or polymer–nanoparticle mixing, often coupled with *in situ* gelation or crosslinking. The synthesis strategies for hybrid hydrogels are diverse and can be divided into physical and chemical crosslinking methods. Physical crosslinking relies on environmental triggers such as temperature, pH, or ionic strength, as well as non-covalent interactions, such as hydrogen bonding and ionic interactions. These methods can yield hydrogels under mild conditions but often result in materials with lower mechanical stability. In contrast, chemical crosslinking involves covalent bond formation through techniques such as radical polymerization, photo-initiated reactions, or the use of crosslinking agents like glutaraldehyde. Advanced chemical approaches include click chemistry (e.g., Thiol-ene, Azide-alkyne cycloaddition), which allows for precise and efficient network formation with high biocompatibility (Majid et al., 2020). Additional strategies such as self-assembly of peptides or proteins, irradiation (UV, electron beam, or gamma), and incorporation of nanoparticles further expand the design possibilities, enabling the fabrication of hydrogels with tailored mechanical, biological, and functional properties.

In biomedical applications, hybrid hydrogels are used as scaffolds for tissue engineering, supporting cell growth and tissue regeneration. They are also applied in drug delivery systems, wound healing, and injectable therapies, where their enhanced mechanical and biological properties are advantageous. Hybrid hydrogels surpass traditional hydrogels in mechanical strength, functional versatility, and biocompatibility, positioning them as promising candidates for next-generation biomedical applications (Marcinczyk et al., 2017; Gholobova et al., 2020).

**Figure 2:**
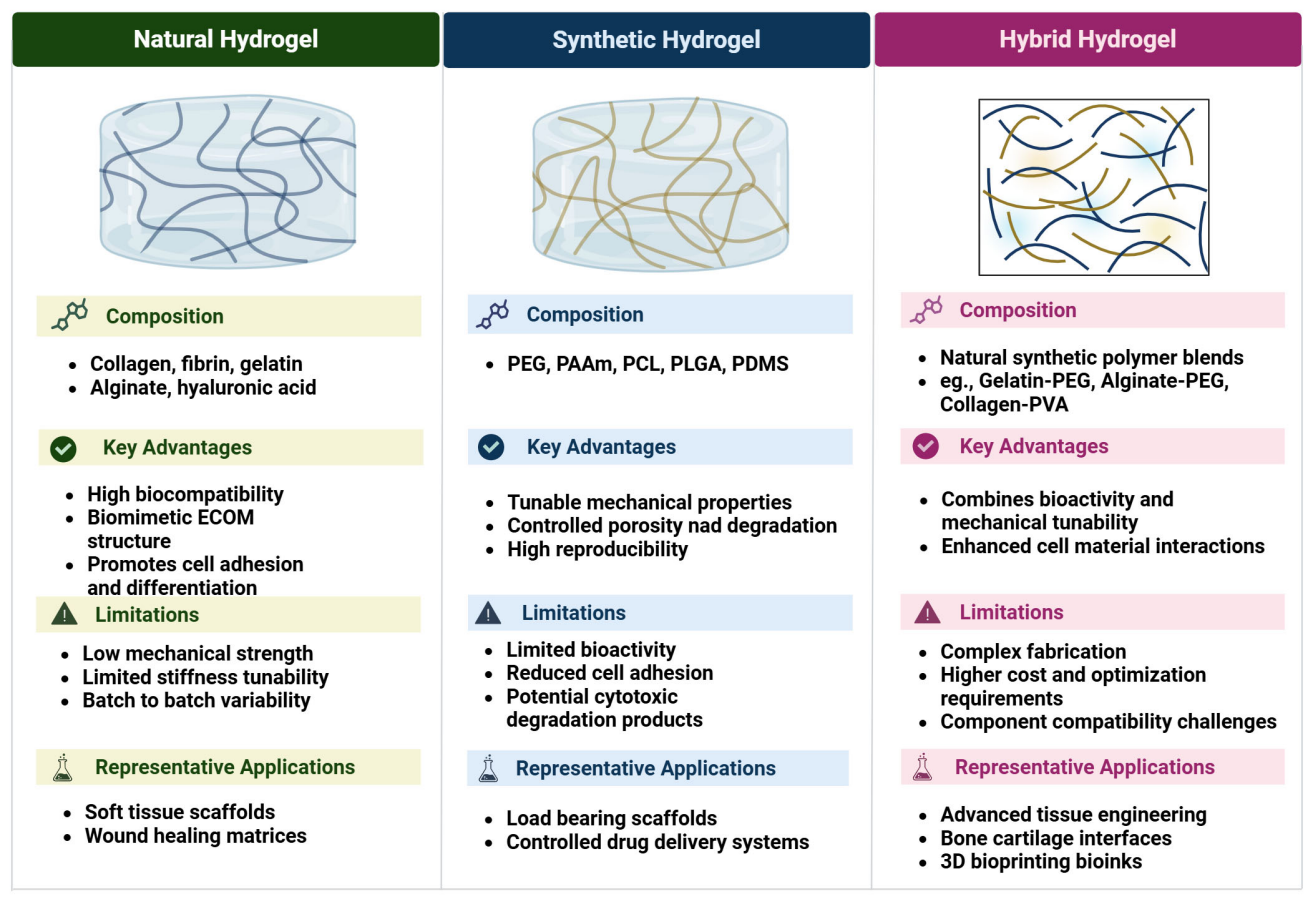
Comparison of natural, synthetic, and hybrid hydrogels used in skeletal muscle tissue engineering, summarizing their typical compositions, key advantages, major limitations, and representative biomedical applications such as soft tissue scaffolds, load−bearing matrices, and advanced bioinks. Created in https://BioRender.com.

**Figure 2** presents a brief overview of the three main types of hydrogels, including their key advantages, limitations, and potential applications. Despite these promising results in the application of hydrogels and soft materials in tissue regeneration, several challenges remain unresolved. Achieving sufficient vascularization and neural innervation is essential for maintaining tissue viability and functional restoration. Recent approaches to host vascularization and host neurons in VML muscle repair involve integrating pre-vasculature and host neurons into the muscle defect site, thereby promoting increased host anastomosis, revascularization, and innervation (García‐Lizarribar et al., 2018). Even in highly vascularized constructs, achieving rapid and stable re−innervation of the graft remains a major bottleneck for restoring coordinated, fatigue−resistant muscle function. Additional obstacles include immunological incompatibility or foreign−body response, the risk of fibrosis or chronic inflammation, and the absence of standardized fabrication protocols and robust translational models, which collectively hinder reproducibility and clinical translation (Chandorkar et al., 2019; Mariani et al., 2019; Zhang et al., 2021). Although many hydrogels are described as biocompatible, both natural and synthetic matrices can still elicit immunological incompatibility and foreign-body reactions (Mariani et al., 2019). For example, impure alginate formulations induce host inflammatory responses and fibrotic encapsulation, whereas highly purified alginate substantially reduces these effects (Otterlei et al., 1991; Kim et al., 2013; Clapacs et al., 2022; Syanda et al., 2022). Likewise, PEG-based hydrogels, often considered bioinert, have been shown to activate complement, recruit macrophages, and promote collagen-rich fibrotic capsules *in vivo*, consistent with a chronic foreign body response (Swartzlander et al., 2015; Jansen et al., 2018). In volumetric muscle loss models, bulk nanoporous hydrogel scaffolds can elicit foreign-body reactions and fibrosis, whereas engineered microporous scaffolds better integrate with host muscle and reduce chronic inflammation.

## 6 Nanoparticles loaded hydrogels

**Figure 3:**
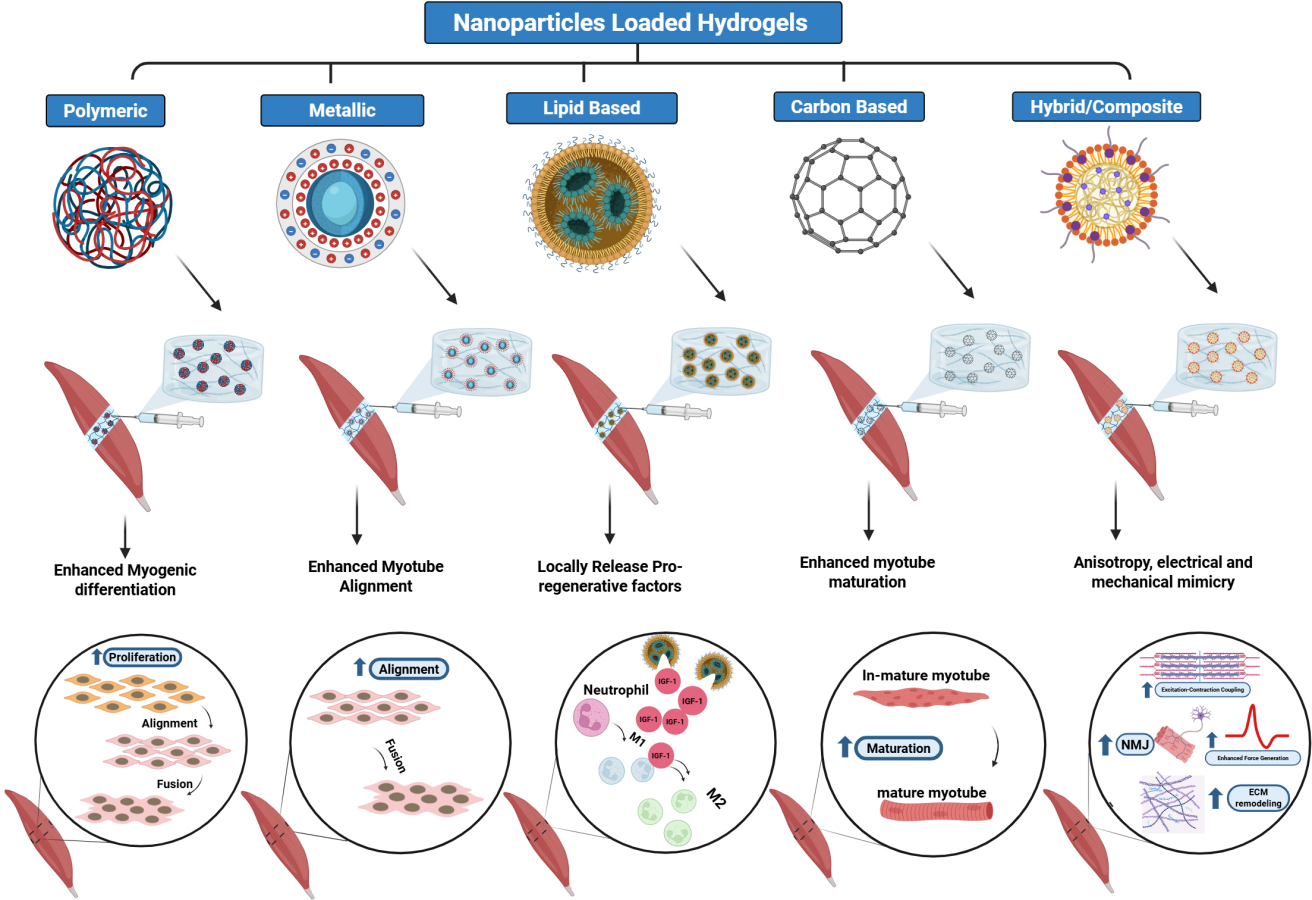
Nanoparticle-loaded hydrogels for treatment of volumetric muscle loss (VML). Schematic overview of nanoparticle−loaded hydrogels for skeletal muscle regeneration, highlighting how different nanoparticle classes-polymeric, metallic, lipid-based, carbon-based, and hybrid/composite-modulate myogenic processes by enhancing myogenic differentiation, myotube alignment, local release of pro−regenerative factors, myotube maturation, and, in the case of hybrid/composite systems, providing anisotropic electrical and mechanical cues that support excitation–contraction coupling, neuromuscular junction formation, and extracellular matrix remodeling. Created in https://BioRender.com.

To address the limitations of pure hydrogels, researchers are increasingly integrating nanoparticles (NPs) into hydrogel matrices to create nanocomposite hydrogels. These composite systems can deliver bioactive signals, enhance mechanical and electrical properties, and modulate the immune microenvironment. The following sections and **Figure 3** discuss the principal subclasses of nanoparticles incorporated into hydrogels for muscle regeneration, including polymeric, metallic, lipid-based, carbon-based, and hybrid nanomaterials, highlighting their distinct functionalities, applications, and translational challenges (Jeong et al., 2022; Han et al., 2023).

### 6.1 Polymeric nanoparticles: bioactive and structural modifiers

Polymeric nanoparticles (NPs) are among the most extensively studied for muscle regeneration because of their biodegradability, biocompatibility, and stimuli-responsive properties. They can be derived from natural polymers (collagen, fibrin, hyaluronic acid, alginate, chitosan) or synthetic ones (polyvinyl alcohol, polyethylene glycol, polylactic acid). Natural polymers mimic the biochemical cues of the ECM, while synthetic materials offer mechanical tunability but often lack inherent bioactivity. Studies have demonstrated the efficacy of polymeric NP-hydrogel systems in controlled drug delivery and bioactive reinforcement. For example, Lee et al. (2021) engineered a thermosensitive Poloxamer 407 hydrogel containing nanoparticles encapsulating CD146 and IGF-1, which locally released pro-regenerative proteins that enhanced satellite cell differentiation and reduced inflammation (Lee et al., 2021). Similarly, Huang et al. utilized PLGA–PEG nanoparticles functionalized with a muscle-targeting peptide to deliver a PTEN inhibitor, resulting in improved muscle growth and regeneration *in vivo (*Huang et al., 2020; Ran et al., 2020; Ge et al., 2021; Raimondo and Mooney, 2021; Han et al., 2023).

Beyond biochemical modulation, polymeric NPs also impart structural benefits. Polydopamine (PDA) nanoparticles, valued for their adhesive and antioxidant properties, were incorporated into an injectable Pluronic hydrogel, significantly improving muscle regeneration and contractile recovery in VML models (Jeong et al., 2022). Another approach involved dextran-aldehyde/gelatin microparticles forming a wet-adhesive hydrogel patch that enhanced muscle healing, reduced fibrosis, and increased angiogenesis (Lee et al., 2024). Electrically conductive polymeric NPs like PEDOT have further expanded the functionality of hydrogels. When combined with GelMA in 3D bioprinted scaffolds, PEDOT (poly-3,4-ethylenedioxythiophene) NPs facilitated electrical conductivity, promoting proliferation, cytoskeletal organization, myotube alignment and myogenic differentiation under electrical stimulation (Wang et al., 2021). This strategy effectively mimics the native bioelectrical environment of muscle tissue and represents a promising avenue for bioelectrically guided muscle regeneration.

A challenge for polymeric NP-hydrogel systems is ensuring controlled, reproducible release kinetics of therapeutic agents. If release is too rapid, the benefits may be short-lived; if too slow or incomplete, regeneration could be suboptimal. Additionally, many studies use immortalized myoblast cell lines (e.g., C2C12) for *in vitro* evaluation, which may not fully replicate primary muscle stem cell behavior (Jeong et al., 2022). Biocompatibility of degradation byproducts is another consideration; while polymers like PLGA degrade to innocuous acids, others (or their crosslinkers) may induce local toxicity or immune responses. So far, polymeric NP hydrogels for muscle have been tested in small animals and scaling up to larger defects and translating them to human muscle injuries remain open challenges.

### 6.2 Metallic nanoparticles: conductivity and biophysical cues

Metallic nanoparticles, including gold (Au), silver (Ag), and iron oxide (Fe_3_;O_4_), are commonly incorporated to introduce electrical conductivity, mechanical reinforcement, and redox activity. Skeletal muscle is an electrically excitable tissue; thus, conductive scaffolds can enhance myotube formation and function. Gold nanoparticles (AuNPs) have been used to create conductive scaffolds that enhance myotube alignment and signal transduction. In a mouse VML model, Ge et al. reported that an injectable AuNP-loaded Pluronic hydrogel improved muscle regeneration and functional recovery. Similarly, gold nanowires (one-dimensional nanostructures) have been used to impart anisotropy and conductivity (Ge et al., 2021). Kim et al. simultaneously 3D-bioprinted gold nanowires and muscle cells in a collagen hydrogel; upon applying an electric field, the nanowires aligned in parallel, guiding myotubes to align and differentiate along the field (Kim et al., 2025). When implanted in a VML model, the aligned Au nanowire–collagen construct showed improved, organized muscle regeneration and decreased fibrotic scar formation relative to non-aligned scaffolds. These findings underscore that metallic nanomaterials can replicate some electrophysiological cues of muscle, promoting structural and functional integration of new fibers.

Silver nanoparticles (AgNPs) provide antimicrobial properties and have also been used to crosslink hydrogels via redox chemistry. For instance, AgNPs complexed with plant-derived lignin were used to create a redox-crosslinked adhesive hydrogel that is tough and self-healing (Namjoo et al., 2023). While that 2019 study was a general proof-of-concept, the implication is that AgNP-incorporated hydrogels could help seal muscle wounds and prevent infection during regeneration. However, one must balance AgNP concentration to avoid cytotoxicity to muscle cells (Lin et al., 2019).

Magnetic iron oxide nanoparticles (e.g., Fe_3_O_4_) represent another subclass with unique utility: they enable remote alignment of scaffolds via magnetic fields. In 2024, Rossi et al. demonstrated that adding magnetic iron oxide particles to an injectable hydrogel allowed the construct to form anisotropic fiber alignments under an external magnetic field, mimicking the parallel orientation of native muscle fibers (Rossi et al., 2025). Such *in situ* magnetically aligned hydrogels showed enhanced myogenic differentiation and regeneration *in vivo*, improved aligned myofiber formation compared to non-aligned controls in preliminary studies. Magnetic NP–NP-hydrogel systems thus offer a contact-free method to induce muscle-like architecture in scaffolds prior to or even after implantation. This strategy addresses a key challenge in muscle tissue engineering: achieving aligned, elongated myofibers that can contract effectively. Magnetic nanoparticles can also be used structurally to control the internal architecture of hydrogels. A 2024 study developed an injectable ECM-mimetic hydrogel composed of collagen type I, hyaluronic acid, and iron oxide magnetic nanoparticles (MNPs). When exposed to a low-intensity static magnetic field, the MNPs aligned collagen fibers into anisotropic bundles, mimicking native muscle architecture. This alignment promoted myotube formation, vascularization, and organized tissue regeneration in a rat tibialis anterior defect model. Such structurally tuned magnetic hydrogels represent a promising approach to guide spatial cell behavior and enhance muscle repair (Griveau et al., 2024).

Metallic NPs can present biocompatibility and safety concerns. While gold is largely inert and well tolerated in the above studies, silver NPs can be cytotoxic at higher doses and may elicit unwanted immune responses. Rigorously optimizing NP concentration is crucial. Iron oxide NPs are generally biocompatible, but they can interfere with MRI or cause local heating under certain conditions. Moreover, metal NPs are typically non-degradable; for example, gold and silver may persist indefinitely, raising questions about long-term fate (though some <10 nm AuNPs can be cleared renally). No clinical studies have yet tested metal-NP hydrogels in patients, and translation will require assurance that metal NPs do not accumulate to harmful levels. Finally, creating uniform dispersions of metallic NPs in hydrogels can be challenging due to aggregation; robust methods (surface functionalization of NPs) are needed to maintain homogeneity and reproducibility.

### 6.3 Lipid-based nanoparticles: biomimetic delivery systems

Lipid-based nanoparticles, such as liposomes, solid lipid nanoparticles (SLNs), and extracellular vesicles (EVs), serve as biocompatible carriers for controlled delivery of growth factors, nucleic acids, or regulatory proteins. While underexplored in muscle tissue engineering, their demonstrated success in wound healing and bone regeneration highlights their potential (Binaymotlagh et al., 2024; Sanati and Amin Yavari, 2024). Hydrogel-integrated liposomes could locally release pro-regenerative factors (e.g., IGF-1, anti-myostatin agents) or immunomodulatory signals that drive macrophage polarization toward pro-healing phenotypes (Raimondo and Mooney, 2021). Of special relevance are EVs, particularly mesenchymal stem cell-derived exosomes, which transfer microRNAs and proteins that stimulate myogenesis (Han et al., 2023). Exosome-laden hydrogels achieve prolonged local activity and protection from degradation, as shown by Ran et al. (2020), who engineered exosomes with muscle-targeting peptides and myostatin inhibitors to enhance regeneration in dystrophic muscle (Ran et al., 2020).

Lipid nanoparticles, while biocompatible, face challenges in vesicle stability and targeting. Unmodified liposomes may diffuse out of a hydrogel or be cleared by immune cells before delivering their cargo. Thus, hydrogel integration must ensure the vesicles remain entrapped until they release their contents. There is also an issue of scale-up and manufacturing consistency; liposomal formulations can be sensitive to production methods, and exosome yields from cells are inherently limited. Moreover, clinical translation of EV-based therapies is still in early stages; questions remain about dosing, immunogenicity (especially if from allogeneic sources), and storage. Nonetheless, lipid-based NP systems represent a promising avenue, particularly for delivering complex biological cues (such as mRNA, microRNA, or protein therapeutics) to regenerate muscle in a controlled manner.

### 6.4 Carbon-based nanoparticles: electroconductive and structural enhancers

Carbon-based nanomaterials, including graphene, graphene oxide (GO), reduced GO (rGO), and carbon nanotubes (CNTs), impart excellent mechanical strength and electrical conductivity to hydrogels. By uniting organic, inorganic, and polymeric nanomaterials, these systems can simultaneously address several challenges in skeletal muscle regeneration, such as oxidative stress, bacterial infection, impaired conductivity, and structural anisotropy, while promoting myogenic differentiation and tissue integration.

These conductive scaffolds not only enhance myoblast proliferation but also facilitate myotube contraction under electrical stimulation *in vitro* (Zhu et al., 2023b). Zhao et al. (2022) developed a gelatin/reduced GO cryogel mimicking the muscle’s elastic and conductive environment, which significantly enhanced regeneration in rat VML models. This study underlined the synergy of combining a biocompatible polymer (gelatin) with a conductive carbon nanofiller (rGO), yielding a scaffold that not only bridged the defect but also electrically and mechanically stimulated muscle regeneration (Zhao et al., 2022).

CNT-based composites also promote uniaxial alignment and conductivity. Ramón-Azcón et al. aligned CNTs in GelMA hydrogels via dielectrophoresis, resulting in improved myogenic marker expression in C2C12 cells (Ramón-Azcón et al., 2013). Hu et al. integrated polydopamine-coated CNTs into a gelatin cryogel, promoting muscle fiber maturation and vascularization (Hu et al., 2022). The resulting conductive, mechanically robust cryogel promoted markedly better muscle regeneration in a rat TA muscle defect, evidenced by more mature muscle fibers and blood vessels in histology. This benefit was attributed to the enhanced mechanical strength and electrical conductivity of the CNT-laden cryogel, which facilitated muscle cell communication and myogenic signal transduction in the regenerating tissue. However, biocompatibility concerns persist, since pristine CNTs and high-concentration graphene materials may induce granulomatous inflammation or resist degradation (Zhu et al., 2023b). Functionalization and dispersion control remain key to mitigating these risks.

### 6.5 Hybrid nanoparticle systems: multifunctional and responsive designs

Hybrid nanocomposite hydrogels combine multiple nanoparticle types to integrate complementary properties. By uniting organic, inorganic, and polymeric nanomaterials, these systems can simultaneously address several challenges in skeletal muscle regeneration, such as oxidative stress, bacterial infection, impaired conductivity, and structural anisotropy, while promoting myogenic differentiation and tissue integration.

#### 6.5.1 Multifunctional hybrid nanocomposites

As a multifunctional hybrid hydrogel, Au/PDA combines ultrasmall gold NPs (inorganic, conductive) and polydopamine NPs (organic/polymeric, antioxidant, and adhesive) within a Pluronic F127 hydrogel matrix. This multifunctional design enabled the scaffold to mitigate oxidative stress, reduce the risk of infection, and restore electrical signaling in injured muscle tissue. In a full-thickness rat muscle defect model, the Au/PDA nanocomposite hydrogel accelerated muscle tissue formation and improved muscle force production and electrophysiological response compared to controls. Furthermore, oriented channels were engineered in this hydrogel via controlled cryogelation to mimic muscle’s anisotropy, demonstrating how hybrid systems can incorporate biophysical cues in addition to biochemical and electrical ones. Similarly, PDA-chelated carbon nanotube–iron oxide (PFeC) nanohybrids embedded in a GelMA/HAMA hydrogel created a magnetically aligned and conductive scaffold. This system promoted myogenic differentiation and vascularization *in vivo*. When supplemented with tryptophan (Trp), the composite hydrogel (HM/GM/PFeC/Trp) further enhanced C2C12 myoblast alignment and differentiation both *in vitro* and in a rat tibialis anterior VML model. The hybrid scaffold reduced inflammation, upregulated myogenic markers (e.g., MyoD, MHC), and promoted robust tissue regeneration. Collectively, these results illustrate the synergistic potential of integrating polymeric, metallic, and carbon nanomaterials to more closely replicate the complex biochemical and biomechanical environment of native skeletal muscle.

#### 6.5.2 Conductive hybrid systems via 3D bioprinting

Another promising hybrid strategy involves using 2D MXene nanosheets (Ti_3_;C_2_T_x_) within a GelMA-based bioink to produce conductive scaffolds via 3D bioprinting (Binaymotlagh et al., 2024). MXenes provided excellent electrical conductivity, mechanical reinforcement, and surface functionality that support C2C12 myoblast proliferation, alignment, and differentiation. The hybrid bioink exhibited elevated expression of myogenic markers (MyoD, myogenin, and MyHC) and facilitated the formation of contractile myotubes *in vitro*. This approach highlights MXene’s electroactive properties and demonstrates the potential for scalable, spatially controlled biofabrication of skeletal muscle tissue constructs using composite nanomaterials.

#### 6.5.3 Environment-responsive hybrid nanoparticles

An emerging direction involves microenvironment-responsive nanocomposites that dynamically respond to pathological cues such as oxidative stress or altered pH (Qi et al., 2025). Recent studies have developed reactive oxygen species (ROS)-responsive hydrogels containing MnO_2_ nanoparticles and polydopamine (PDA) within a hyaluronic acid-based matrix. In models of muscle atrophy and chronic injury, these MnO_2_@PDA nanozymes scavenged ROS and modulated inflammation, downregulating markers of muscle degradation and pro-inflammatory cytokines. Such stimuli-responsive systems provide “on-demand” therapeutic action, releasing or activating bioactive components in response to disease-associated biochemical signals.

#### 6.5.4 Multimodal hybrid therapies

Hybrid systems can also integrate biomaterial scaffolds with cell or growth-factor therapies. For example, VEGF-loaded polymer nanoparticles embedded within fibrin hydrogels containing muscle progenitor cells have been shown to promote both myogenesis and angiogenesis *in vivo (*Zhu et al., 2023b). Another approach combines decellularized muscle matrix (biochemical specificity) with graphene nanosheets (electrical conductivity) in a scaffold, illustrating the potential of bio–nano hybrids that merge biological cues with nano-enabled functionality. While still emerging, these combinatorial systems hold promise for recreating the multifaceted regenerative environment of native muscle tissue. **Table 1** below summarizes the various nanoparticles and their functions with examples and applications in the soft tissue regeneration field.

**Table 1:** Summary of various nanoparticles and their functions with examples and applications in the soft tissue regeneration field.

Nanoparticle type	Function	Example materials	Applications/Results
Polymeric	Drug delivery, antioxidant support, and mechanical reinforcement	PLGA (Yacoub et al., 2022; El Hoffy et al., 2023), PEG (Griveau et al., 2024), PDA (Binaymotlagh et al., 2024), Poloxamer, GelMA (Kim et al., 2022; Awad et al., 2025)	Enhanced myogenic differentiation, bioadhesion, and ROS scavenging
Metallic	Conductivity, magnetic alignment, and antimicrobial action	Gold (AuNPs), Silver (AgNPs), Iron Oxide (Fe_3_O_4_) (Greising et al., 2017; Hu et al., 2022)	Myotube alignment, antimicrobial hydrogels, magnetically guided regeneration
Lipid-Based	Bioactive delivery (e.g., growth factors, RNAs), anti-inflammation	Liposomes (Yacoub et al., 2024), Exosomes (Moon and Chang, 2022), Solid Lipid Nanoparticles (SLNs) (Ashin et al., 2024; Aziz et al., 2024)	Localized sustained release, macrophage polarization, targeted repair
Carbon-Based	Electrical conductivity, mechanical strength	Carbon Nanotubes (CNTs), Graphene Oxide (GO), rGO (Ramón-Azcón et al., 2013; Jo et al., 2017)	Myotube maturation, enhanced conductivity, guided alignment
Hybrid/Composite	Multi-functionality: combines mechanical, electrical, and biochemical cues	Au+PDA, PDA-CNT-Fe_3_0_4_ MXene- GelMA, ATP-PCL-PEG (Chen et al., 2021; Hemmati et al., 2021)	Anisotropy, electrical and mechanical mimicry, environment-responsive systems

## 7 Cellularized hydrogels for tissue regeneration

Cellularized hydrogels are three-dimensional (3D) polymer networks that encapsulate living cells within a matrix designed to emulate the structure and biochemical cues of the native extracellular matrix (ECM) found in tissues. ECM is crucial for providing both support and functional signaling to surrounding cells, creating an optimal environment for cell proliferation, differentiation, and tissue homeostasis. Unlike traditional two-dimensional (2D) cell culture systems, cellularized 3D hydrogels offer a more physiologically relevant model, enabling more precise control of cell fate and function while enabling nuanced investigation of cell-matrix interactions.

The formation of cellularized hydrogels typically involves first preparing a hydrogel precursor from functional polymers capable of crosslinking, such as polyethylene glycol (PEG) acrylates, Gelatin methacrylates (GELMA), or hyaluronic acid derivatives. Living cells are harvested, counted, and uniformly suspended within the hydrogel precursor solution before gelation begins. This step ensures even cell distribution throughout the resulting 3D scaffold. Gelation may be induced via photo polymerization, chemical cross-linking, or temperature-responsive methods. Once solidified, the hydrogel is cultured in appropriate media, providing a hydrated, porous matrix that allows nutrients and gases to diffuse in and waste to diffuse out.

Importantly, the porosity and degradability of cellularized hydrogels are highly tunable, enabling dynamic remodeling by encapsulated cells and supporting processes such as cell proliferation, migration, and differentiation. Researchers can assess cell behavior within the hydrogel, such as viability, morphology, proliferation, differentiation, and gene expression, using advanced microscopy techniques, biochemical assays, and live imaging strategies (Khetan and Burdick, 2009). Numerous studies have shown that culturing muscle progenitor or stem cells within proper hydrogel matrices enhances myogenic differentiation and functional tissue regeneration *in vitro* and *in vivo*, especially when using biomimetic materials such as decellularized ECM-derived hydrogels (Ungerleider et al., 2020).

By providing key mechanical, structural, and compositional elements of the native ECM, hydrogels serve as powerful tools for investigating morphogenesis, aging, and disease pathology (Caliari and Burdick, 2016). By mimicking the cellular organization and functionalities found in human tissues, 3D cultures provide superior platforms for studying complex conditions, including muscular dystrophies, cancer, and neurodegenerative disorders (Park et al., 2024). 3D cultures also play a crucial role in drug discovery by enabling researchers to assess the safety and efficacy of potential drugs. Recent research demonstrates that tissue-specific ECM hydrogels, especially those derived from skeletal muscle, can further enhance regenerative outcomes by providing an optimal niche for satellite cell proliferation and differentiation and by reducing fibrotic responses after injury (Barajaa et al., 2025). The following section will showcase various engineered cellular microenvironments and the functional dimensions of 3d hydrogel encapsulation.

### 7.1 Three-dimensional cell encapsulation and biomimicry

Cells can be embedded within hydrogels by mixing a cell suspension into the hydrogel precursor before gelation. Gelation, triggered by physiological temperature, light, or crosslinking chemistry, encapsulates cells evenly throughout the 3D matrix. Once the gel forms, typically at physiological temperature (e.g., 37 °C), cells are evenly distributed throughout the 3D matrix. In this setup, cells are suspended in a liquid hydrogel precursor, evenly distributed, and then gelled by triggers such as temperature changes, light exposure, or chemical crosslinking, all under conditions compatible with cell viability. This process produces a 3D culture system in which cells experience interactions and physical cues similar to those found in native tissues, which influences morphology, proliferation, differentiation, and gene expression. In 3D cell cultures, cancer cells are grown in an environment that closely mimics the 3D architecture and complexity of *in vivo* tumors. This approach has revolutionized cancer research by providing a more accurate representation of the tumor microenvironment (TME) and enabling the study of tumor behavior and response to therapies in a more physiologically relevant context (Abuwatfa et al., 2024). 3D encapsulation in hydrogels has significantly enhanced our understanding of cell biology, revealing behaviors and therapeutic outcomes not observable in traditional cell culture systems.

### 7.2 Cell morphology and function

The extracellular matrix’s composition and physical properties within hydrogels direct integrin signaling and cytoskeletal organization, thereby altering gene expression and cell function, reflecting physiological conditions (Kim et al., 2019). In 3D hydrogels, cells exhibit distinct morphologies, such as rounded shapes and cluster formation, in contrast to the stretched, flattened morphology seen on 2D surfaces. This difference closely mirrors the structure and organization observed *in vivo*. Specifically, studies have shown that in 3D matrices, cells form spheroids, display enhanced cell–cell contacts, and exhibit cytoskeletal organization that differs from that seen in 2D cultures (Huang et al., 2013; Kang et al., 2021).

### 7.3 Protection and mechanical support

The hydrogel matrix protects cells from shear-induced injury during injection or transplantation by buffering mechanical force and maintaining membrane integrity. Elevated levels of shear stress can disrupt cell membranes and cause significant cell death. Encapsulating cells within hydrogels provides mechanical protection from shear forces, helping maintain membrane integrity and enhancing cell survival, particularly in transplantation and regenerative medicine applications. Shear-thinning, injectable hydrogels are engineered to shield cells from mechanical damage during delivery by absorbing and dissipating shear forces encountered during injection, while simultaneously promoting cell viability and engraftment at the target site. Comprehensive reviews on hydrogels for cell and therapeutic applications note that hydrogels act as a barrier to mechanical insults and maintain local cell viability in hostile environments by limiting direct exposure to mechanical and shear stress (Lu et al., 2024). This configuration allows cells to interact with their surroundings in all directions, resembling their behavior *in vivo*. The viscoelastic, stress-relaxing nature of hydrogels allows them to deform in response to physical stress, thereby distributing and minimizing mechanical loads on encapsulated cells. This mechanical buffering further enhances cell survival, spreading, and function (Chaudhuri et al., 2016).

### 7.4 Dynamic microenvironments

Dynamic behaviors within hydrogels can be engineered to recapitulate the native extracellular matrix complexity, allowing cells not only to survive but also to remodel their surroundings, migrate, proliferate, and differentiate – crucial processes in development, regeneration, and disease. Engineered hydrogels often incorporate degradable crosslinks that respond to cell-secreted enzymes, such as matrix metalloproteinases (MMPs), enabling encapsulated cells to locally erode and reorganize the matrix by extending lamellipodia and filopodia, thereby supporting cell spreading and migration. For example, polyethylene glycol (PEG)-based hydrogels engineered with MMP-sensitive peptides or hydrolytically labile ester bonds demonstrate controlled degradation profiles that align temporally with cellular remodeling demand. The degradation rate is tunable by adjusting the peptide sequence, crosslink density, and molecular weight of the PEG macromers. Such remodeling promotes cell migration, proliferation, and spreading as cells locally degrade the matrix, which is linked directly to the density and sensitivity of cross-links (Hoffman et al., 2014). Beyond degradability, dynamic mechanical properties of hydrogels have emerged as powerful regulators of cell behavior. Unlike static substrates, hydrogels with tunable stiffness or viscoelasticity can mimic the time-dependent changes in tissue mechanics seen during healing and disease progression (Jeon et al., 2021). Recent studies highlight a dynamic, controllable 3D biomimetic hydrogel model formed by a dual-functional gelatin macromer that can form a double-network via sequential enzymatic and light-induced crosslinking. This system initially enabled mesenchymal stem cells (MSCs) to spread in a compliant matrix, but they experienced increased stiffness after secondary crosslinking. Late-stage stiffening significantly enhanced osteogenic outcomes, including ECM secretion, osteogenic gene expression, and nuclear localization of YAP/TAZ. *In vivo* studies further demonstrated that dynamic stiffening promoted MSC-mediated bone remodeling, highlighting the role of 3D dynamic stiffening as a key biophysical cue regulating stem cell fate and enhancing bone regeneration (Li et al., 2023a). Photodegradable hydrogels further enable spatial and temporal control, in which light irradiation increases or decreases the crosslink density in polymer matrices by incorporating specific photosensitive compounds (Zhu et al., 2020). The local softening, erodible environment for cells allows researchers to dissect how cells sense and adapt to dynamic changes in their immediate microenvironment. Additionally, the gradual erosion of hydrogels by enzymatic action creates evolving mechanical heterogeneity, facilitating complex morphogenetic processes during tissue development and repair (Lalitha et al., 2017). The combination of degradability, stimuli responsiveness, and mechanical dynamics in hydrogels constitutes a sophisticated biomaterial platform that enables control over cellular microenvironments, thereby enabling functional tissue regeneration and advanced *in vitro* disease modeling. These dynamic features are key to bridging the gap between engineered constructs and the adaptive nature of native tissues.

### 7.5 Seeded cells for skeletal muscle tissue engineering

Seeded cells serve as the foundation of SMTE as they are the fundamental biological units introduced into biomaterial scaffolds, such as hydrogels, to initiate and regulate tissue regeneration and functional restoration. These cells act as living building blocks, capable of anchoring to the scaffold matrix, proliferating, migrating, and undergoing lineage-specific differentiation within a three-dimensional environment, thereby recapitulating key aspects of native muscle tissue repair and formation. The success of engineered constructs largely hinges on the cells’ ability to interact dynamically with both the scaffold and their surrounding microenvironment, responding to physical, chemical, and biochemical cues to influence survival, integration, maturation, and long-term functional performance after implantation. Beyond repopulating the scaffold, seeded cells can secrete an array of trophic factors, cytokines, and extracellular matrix components that actively remodel the local matrix, recruit host cells, promote angiogenesis, modulate inflammation, and restore tissue function (Xiang et al., 2024). The interplay between these cells and the engineered microenvironment determines not only how well the tissue regenerates, but also how rapidly and robustly it integrates with the host (Lumelsky, 2021). As such, the careful selection, sourcing, and conditioning of seeded cells, along with optimization of scaffold design and culture conditions, remain critical determinants of therapeutic success in skeletal muscle tissue engineering. In preclinical VML models, several studies have directly tested cell−laden constructs for functional repair (Corona et al., 2016). Keratin hydrogels loaded with myogenic cells and growth factors improved muscle mass and force production in a mouse VML defect compared with acellular controls (Baker et al., 2017). Fibrin microthread scaffolds seeded with adult human cells, and related growth-factor-releasing constructs, enhanced *de novo* myofiber formation and restored contractile output in rodent VML injuries (Page et al., 2011; Grasman et al., 2015b; Grasman et al., 2015a). Vascularized, myoblast−seeded scaffolds and engineered 3D muscle tissues have further promoted revascularization, innervation, and force recovery following implantation in animal VML models (Gilbert‐Honick and Grayson, 2020). Collectively, these studies illustrate how the choice and organization of seeded cells critically determine the regenerative efficacy of biomaterial−based therapies for VML (Qi et al., 2025).

#### 7.5.1 Satellite cells

Satellite cells (SCs) are the principal muscle stem cells responsible for skeletal muscle regeneration and maintenance. Often found in higher density near neuromuscular and myotendinous junctions. SCs express transcription factor paired box 7 (Pax7), which is essential for their maintenance, survival, and function. Pax7 acts as a master regulator controlling gene expression programs that maintain satellite cells in a quiescent, stem-like state and regulate their activation and proliferation upon injury or stress (Sincennes et al., 2021). In quiescent SCs, Pax7 promotes expression of inhibitors of differentiation such as Id2 and Id3, which block premature myogenic differentiation and preserve the stem cell pool (Kumar et al., 2009). During activation, Pax7 continues to regulate the expression of myogenic regulatory factors such as Myf5 and MyoD, balancing proliferation and differentiation. Pax7 also inhibits differentiation by repressing MyoD-dependent activation of myogenin, thereby tightly controlling the timing of muscle regeneration (Olguin and Olwin, 2004). Upon muscle damage, exercise, or other stress stimuli, SCs are rapidly activated. Once activated, SCs re-enter the cell cycle, proliferate as myoblasts, and differentiate into multinucleated myotubes that either regenerate new muscle fibers or fuse with existing fibers to repair and strengthen muscle tissue (Kaczmarek et al., 2021). Satellite cells also contribute to postnatal muscle growth and adaptation by donating additional myonuclei to muscle fibers under increased mechanical load (exercise-induced hypertrophy), thereby enabling greater protein synthesis and muscle function (Snijders et al., 2015). However, aging and chronic diseases lead to muscle loss and dysfunction, a condition called sarcopenia, which is characterized by a reduction in SC number and function. Despite their advantages, SCs present several challenges for therapeutic application. They exhibit limited proliferative capacity *in vitro* and rapidly lose quiescence and myogenic potential once removed from their native niche, often undergoing premature differentiation (Cosgrove et al., 2009). This niche dependence hinders large-scale expansion, making it difficult to generate sufficient functional cells for transplantation (Syverud et al., 2014). Consequently, alternative cell sources, such as mesenchymal stem cells (MSCs), induced pluripotent stem cells (iPSCs), and other progenitor populations, are increasingly being explored to overcome these limitations and enhance skeletal muscle tissue engineering outcomes.

#### 7.5.2 Myoblasts/C2C12 cells

The C2C12 cell line is one of the most widely used and well-characterized models for studying skeletal muscle biology, myogenic differentiation, and muscle-related diseases *in vitro.* Originating as a subclone from the primary cultures of adult C3H mouse leg muscle established after injury, C2C12 myoblasts exhibit a classic spindle-shaped, myoblast-like morphology and rapid proliferation rate, features ideal for controlled experimental manipulation. Differentiated C2C12 myotubes demonstrate contractility and express multiple isoforms of muscle contractile proteins, including cardiac and skeletal troponin T, making them a useful model to study muscle function and calcium sensitivity (McMahon et al., 1994). Under growth conditions, C2C12 cells proliferate as undifferentiated myoblasts. Still, when subjected to low-serum differentiation media, they efficiently fuse into multinucleated myotubes that resemble native muscle fibers both morphologically and molecularly. During differentiation, expression of key myogenic markers such as Myogenin (MYOG), Myosin Heavy Chain (MYH2), and structural proteins increases, accompanied by clear sarcomeric organization within the engineered myotubes (Zhang et al., 2021). This makes C2C12 a cornerstone for analyzing signaling pathways, gene regulation, and the functional maturation of muscle. C2C12 cells have also been employed in three-dimensional (3D) engineered tissues to study their interactions with different scaffolds, growth factors, and the microenvironment. Electrical stimulation, substrate composition, and topographical cues can further modulate the maturation of C2C12-derived muscle constructs, facilitating analysis of how functional properties such as contractility develop under distinct conditions (Cao and Warren, 2025). C2C12 cells are widely used as a versatile *in vitro* model for muscle biology research, providing invaluable insights into myogenesis, muscle differentiation, and regeneration. They are widely used to study the cellular and molecular mechanisms underlying muscle development, specifically enabling detailed investigation into the regulation of gene expression, transcriptional networks, and cellular signaling during myoblast fusion and myotube formation. C2C12 cells serve as a reliable model for drug screening and toxicity studies, enabling researchers to assess how candidate therapeutics and compounds influence muscle cell metabolism, differentiation, and function, an essential step in developing treatments for muscle-related disorders. Furthermore, they serve as disease modeling systems, particularly for muscular dystrophy and muscle wasting, enabling the exploration of pathophysiological features and the testing of potential interventions (Li et al., 2008). However, several limitations exist. As a murine immortalized line, C2C12 cells differ from primary human myoblasts in gene expression, physiological responses, and maturation capacity, limiting direct clinical translation. The C2C12 cell line has a finite lifespan, generally maintaining viability for only 10–50 passages before entering senescence, with successful differentiation typically achievable at earlier passages. Functionally, C2C12 myotubes may exhibit lower contractile stress, show a reduced twitch-to-tetanus ratio, and are more prone to detachment compared to constructs derived from primary or avian myoblasts (Santoso et al., 2021). Moreover, maintaining experimental reproducibility requires careful regulation of cell density, serum conditions, and passage number, as overconfluence or extended culture periods can trigger premature differentiation and introduce substantial variability (McMahon et al., 1994).

#### 7.5.3 Mesenchymal stem cells

Mesenchymal stem cells (MSCs) are adult multipotent stem cells with significant promise for skeletal muscle tissue engineering and regenerative medicine due to their capacity for self-renewal, plasticity, and ability to differentiate into multiple lineages, including myocytes, osteoblasts, chondrocytes, and adipocytes (Jackson et al., 2010). They are attractive for clinical applications because they can be harvested from various tissues, such as bone marrow, skeletal muscle, periosteum, and adipose tissue, and are associated with fewer ethical and legal concerns than embryonic stem cells (Rosenbaum et al., 2008). A distinguishing feature of MSCs is their ability to produce secretory factors that play critical roles in tissue repair, supporting both engraftment and trophic functions (autocrine and paracrine) (Samsonraj et al., 2017). Key secretory factors produced by MSCs include vascular endothelial growth factor (VEGF), hepatocyte growth factor (HGF), insulin-like growth factors (IGF-1 and IGF-2), fibroblast growth factor (FGF), and various interleukins such as IL-6 and IL-10. These factors collectively promote angiogenesis, enhance survival and proliferation of resident muscle stem cells (satellite cells), suppress fibrosis by modulating extracellular matrix remodeling, and regulate immune cell activity to reduce chronic inflammation and scar formation (Han et al., 2022). For example, VEGF couples osteogenesis with angiogenesis by regulating osteoblast and osteoclast function while simultaneously driving new blood vessel formation, thereby enhancing bone development and ensuring efficient oxygen and nutrient delivery essential for tissue repair (Hu and Olsen, 2017). Beyond their direct myogenic differentiation, MSCs support skeletal muscle repair by secreting growth factors, cytokines, and extracellular vesicles that enhance local angiogenesis, modulate inflammatory and immune responses, suppress fibrosis, and promote endogenous muscle cell regeneration (Sandonà et al., 2021). For instance, MSCs contribute to muscle repair by modulating inflammatory responses, particularly by promoting the transition of macrophages from a pro-inflammatory M1 phenotype, which clears cellular debris and pathogens, to an anti-inflammatory M2 phenotype that supports tissue regeneration (Qi et al., 2025).

#### 7.5.4 Adipose-derived mesenchymal stem cells

Among the MSC subtypes, adipose-derived mesenchymal stem cells (AD-MSCs) are notable for their abundance and the ability to provide a viable allogeneic source via minimally invasive harvesting (liposuction), compared with obtaining bone marrow or muscle biopsies (Jackson et al., 2010). AD-MSCs share the defining characteristics of MSCs, plastic adherence in culture, self-renewal, and multipotency, allowing them to maintain a reservoir of undifferentiated stem cells and differentiate into various mesenchymal and non-mesenchymal lineages, including myocytes, osteocytes, chondrocytes, and even neuronal cells under appropriate conditions (Suchanecka et al., 2025). Recent studies have examined the effect of cell culture passage on the therapeutic efficacy of mesenchymal stromal cells. Bone marrow-derived MSCs (BM-MSCs) were harvested from the femur of Wistar rats, while adipose tissue-derived MSCs (AT-MSCs) were isolated from inguinal fat (Carmona-Luque et al., 2024). Across culture passages, BM-MSCs exhibited alterations in both their functional and phenotypic characteristics, which may compromise their effectiveness as a cell therapy treatment. However, AT-MSCs maintained a normal phenotype and preserved their differentiation capacity throughout culture. Moreover, emerging evidence highlights the ability of AT-MSCs to promote myogenic regeneration *in vitro* and *in vivo*, thereby enhancing engraftment, tissue repair, and functional restoration. These effects occur through either direct differentiation into myocytes or via fusion with host cells, using sheep as animal models of muscle injury (Muniz et al., 2024). In addition to direct engraftment, AD-MSCs can potentiate muscle recovery by interacting with and supporting resident satellite cells and fibro-adipogenic progenitor (FAP) populations, either through secreted bioactive molecules or by contributing to the cellular heterogeneity required for optimal muscle healing (Sastourné-Arrey et al., 2023).

#### 7.5.5 Induced pluripotent cells

Induced pluripotent cells (iPSCs) have become increasingly utilized and versatile seed cells in SMTE due to their self-renewal capacity and ability to differentiate into skeletal muscle lineage cells. iPSCs express pluripotency genes (Sox-2, Oct-4, c-Myc, and Klf4) and can be expanded, offering a scalable and potentially autologous cell source for muscle regeneration. IPSCs can differentiate into myogenic progenitor cells (iMPCs) that closely resemble muscle satellite cells in their proliferative potential and capacity for muscle fiber formation. The first strategy employed to achieve these transgenic approaches involves transient overexpression of key muscle-specific transcription factors (Pax7, Pax3, or MyoD), often combined with modulation of signaling pathways using GSK3β inhibitors, such as CHIR99021 or 6-bromoindirubin-3′-oxime, to influence Wnt/beta-catenin signaling (Iberite et al., 2022). Second, a non-transgenic strategy involves mimicking the natural, stepwise process of muscle development. This approach uses iPSC-derived mesodermal cells cultured with a GSK3β inhibitor (CHIR99021) and a BMP inhibitor (LDN193189), followed by exposure to key growth factors such as HGF, IGF-1, and FGF2 (Thiab et al., 2025). By applying selective culture conditions and tailored combinations of these factors, the method aims to faithfully recapitulate *in vitro* the sequential specification and differentiation events characteristic of embryonic myogenesis. While small-molecule differentiation can lead to the formation of multinucleated myotubes, this approach is less efficient and slower, yielding populations that contain non-myogenic cells, which reduces scalability for drug screening or potential therapeutic applications. However, while enabling multilineage differentiation, any residual undifferentiated state of iPSCs poses a risk of teratoma (tumor) formation if transplanted *in vivo (*Gutierrez-Aranda et al., 2010; Lee et al., 2013; Nelakanti et al., 2015). Beyond lineage specification, the maturation of iPSC-derived muscle is strongly influenced by contractile stimulation and pro−differentiation cues (Iberite et al., 2022). Electrical or mechanical stimulation of engineered myobundles enhances myofibrillar organization, hypertrophy, calcium-handling, and force production, indicating a more adult-like contractile phenotype (Khodabukus et al., 2019). When combined with differentiation-promoting factors such as IGF−1, TGFβ pathway inhibitors, or neurotrophic support, contractile stimulation further improves sarcomeric alignment, excitation–contraction coupling, and the ability of iPSC-derived myotubes to generate sustained, tetanic contractions under pacing (Dreher et al., 2024). These findings suggest that integrating dynamic contractile conditioning with biochemical differentiation factors is a key strategy to drive iPSC-derived skeletal muscle from a fetal-like state toward functional maturity suitable for disease modeling and regenerative applications (Iberite et al., 2022).

### 7.6 Drug-loaded hydrogels for skeletal muscle regeneration

Hydrogels have emerged as versatile and powerful carriers for localized delivery of regenerative therapeutics in skeletal muscle repair. Their biocompatibility, adjustable mechanical properties, and minimally invasive delivery make hydrogels a preferred choice for both drug and cell delivery systems in muscle tissue engineering. Drug-loaded hydrogels overcome the limitations of conventional muscle regeneration strategies by facilitating localized, sustained, and tunable release of therapeutics directly at injury sites. These systems support *in situ* gelation, enable targeted administration, and allow for customizable degradation rates—characteristics that promote functional recovery by maintaining prolonged therapeutic levels (Zöller et al., 2025). Their bionic structure further enables encapsulation of a wide range of agents, from small molecules and proteins to nucleic acids and living cells, with spatial and temporal control over regeneration cues. The following is a presentation of the different classes of therapeutic cargoes used in hydrogel systems.

#### 7.6.1 Small molecules

Small molecules are organic substances with low molecular weight (usually less than 1kDa) that can penetrate cells and highly selectively alter intracellular signaling pathways. They are excellent candidates for therapeutic usage in regenerative medicine due to their stability, manufacturability, and decreased. Specifically, when paired with scaffolds such as hydrogels, which allow for regulated, sustained administration to injured tissues, tiny bioactive molecules have demonstrated promise in musculoskeletal regenerative engineering (Calin et al., 2025). Additionally, a strong toolkit for adjusting small-molecule release kinetics is provided by the sensible design of hydrogels, which customizes network mesh size, degradability, and drug–matrix interactions.

Innovative hydrogel systems have enabled the targeted and controlled delivery of small molecules, such as anti-inflammatories, antioxidants, and ions, to sites of skeletal muscle injury. For instance, Rodriguez-Romano et al. discovered that NaBC1 is a mechanosensory that interacts with growth factor receptors (GFRs) and fibronectin-binding integrins to form functional clusters that enhance mechanotransduction and tissue regeneration processes, such as muscle regeneration, adhesion-driven osteogenesis, and angiogenesis. They investigated if borax was released from injectable alginate hydrogels in the quadriceps of symptomatic SOD1G93A mice, a model in which muscle denervation precedes motor neuron loss. It was found that borax-loaded alginate hydrogels demonstrated reduced muscle atrophy and preservation of muscle fiber integrity, with the injectable structure allowing local release and minimizing systemic side (Rodriguez-Romano et al., 2025).

Manganese dioxide (MnO_2_) is a redox-active inorganic compound known for its reactivity with reactive oxygen species (ROS), making it a valuable antioxidant material in biomedical applications. The immobilization of MnO_2_ onto polymer dots creates nanoscale hybrid systems that respond to oxidative conditions in a specific manner. Under typical physiological conditions, these MnO_2_-immobilized polymer dots may scavenge excess ROS while remaining stable due to their ROS-sensitivity (Kim et al., 2025). In injured or inflammatory skeletal muscle tissue, their incorporation into hydrogel matrices supplies persistent antioxidant activity and enables targeted, ROS-triggered phase changes. Expanding on this idea, Kim et al. created an injectable MnO_2_@PD-HGC hydrogel that responds to elevated ROS levels in damaged muscle tissue by undergoing an irreversible sol-to-gel transition (Kim et al., 2025). By embedding MnO_2_-complexed polymer dots (MnO_2_@PD) into a thermoresponsive hexanoyl glycol chitosan (HGC) matrix, the hydrogel was created, allowing for real-time responsiveness through conductivity and fluorescence alterations as well as ROS-triggered structural transformation (Huang et al., 2024). In a mouse model of muscle injury, this system demonstrated long-term antioxidant activity and significantly promoted muscle regeneration, highlighting its potential as a smart, ROS-sensitive therapeutic platform (Li et al., 2022).

It has been determined that ions like magnesium (Mg²^+^) are essential for muscle growth. Magnesium ions are good candidates for inclusion in drug-loaded hydrogels because they affect muscle contractility and support cellular metabolism. By encouraging myogenesis and satellite cell activation, hydrogel systems that release magnesium ions under controlled conditions have shown improved regenerative outcomes (Relaix and Zammit, 2012; Relaix et al., 2021). Chen et al. (2021) developed an injectable hydrogel that self-heals by releasing curcumin and magnesium ions. With a tensile strength of 800 kPa, this hydrogel not only provided mechanical stability but also ensured continuous curcumin release for 21 days. The dual release method was created to encourage the restoration of tendons to bone, and it may be modified to increase cell viability and reduce inflammation, thereby facilitating the regeneration of skeletal muscle (Chen et al., 2021).

Limitations still exist, such as the difficulty of distributing drugs uniformly across vast or irregular muscle lesions and the need to ensure that drug release kinetics properly match the intricate, time-dependent stages of tissue repair (Qi et al., 2025). To maximize the therapeutic efficacy and clinical translation of such hydrogel systems, these problems must be resolved.

#### 7.6.2 Growth factors

Hydrogel-mediated hormone delivery has primarily focused on growth-promoting factors such as insulin-like growth factor-1 (IGF-1), which potently stimulate myogenic differentiation and accelerate skeletal muscle regeneration (Qi et al., 2025). Incorporating IGF-1 into decellularized extracellular matrix (dECM)-derived hydrogels provides a biomimetic microenvironment that enhances muscle precursor cell proliferation and functional recovery following volumetric muscle loss, outperforming conventional bolus injection approaches. This strategy leverages the native biochemical cues of the ECM to guide and sustain tissue regeneration (Barajaa et al., 2025).

Recent studies further underscore the regenerative potential of dECM-based hydrogels. Barajaa et al. (2025) reaffirm that these materials represent a versatile class of biomimetic scaffolds capable of recapitulating essential ECM architecture and signaling, thereby promoting skeletal muscle repair (Barajaa et al., 2025). Similarly, Lei Qi et al. highlight the broader regenerative engineering paradigm that integrates seed cells, scaffolds, and bioactive molecules, and position hydrogel-based IGF-1 delivery as a particularly effective approach within this framework (Qi et al., 2025).

Advanced hydrogel designs incorporating heparinized matrices, affinity motifs (e.g., aptamers), and degradable crosslinks enable the controlled release of IGF-1, VEGF, HGF, and PDGF, coordinating myogenesis and angiogenesis while reducing systemic side effects (Lev et al., 2024; Calin et al., 2025).

Despite these advances, key challenges remain. Maintaining hormone stability and bioactivity within hydrogel matrices and precisely aligning release kinetics with the temporal dynamics of muscle repair are critical to optimizing therapeutic efficacy and translating these strategies into clinical practice (Barajaa et al., 2025; Qi et al., 2025).

#### 7.6.3 Cytokines and Peptides

Hydrogels functionalized with immunomodulatory cytokines (e.g., interleukin-4) and peptides (e.g., SDF-1) have demonstrated diverse, cell–type–specific effects in advancing skeletal muscle regeneration (Ostaszewska et al., 2025). These systems facilitate localized control over the inflammatory environment, promote macrophage polarization toward pro-regenerative phenotypes, and enhance overall tissue repair outcomes in both *in vitro* and animal models (Li et al., 2023b; Ostaszewska et al., 2025). Advanced delivery systems aim to overcome cytokine instability and rapid degradation, necessitating spatial and temporal regulation of release to maximize therapeutic impact.

#### 7.6.4 Extracellular Vesicles (EVs)

Emerging literature highlights extracellular *Vesicles* (EVs), particularly those derived from mesenchymal stem cells, as highly stable, low-immunogenic cargo capable of mediating tissue repair signals (Ju et al., 2023). Encapsulation within hydrogels prevents premature clearance and enables prolonged, localized action. The combination of drugs, cytokines, and EVs has been shown to synergistically improve muscle regeneration via coordinated signaling in injured tissues.

### 7.7 Mechanisms of hydrogel drug release

#### 7.7.1 Diffusion-controlled release

Diffusion is the simplest release pathway, in which concentration gradients drive agents from the hydrogel matrix to the surrounding tissue. Release kinetics are governed by network pore size, crosslinking density, and hydrophilicity.

#### 7.7.2 Stimuli-responsive release

Recent engineering advances have led to the creation of stimuli-responsive hydrogels that are triggered by environmental cues such as pH, temperature, ionic strength, or specific enzymes (e.g., MMP-13). Such systems enable site-specific and on-demand delivery to release therapeutic payloads in pathological microenvironments in response to injury-associated signals (Calin et al., 2025).

#### 7.7.3 Degradation-controlled release

In degradation-mediated systems, the breakdown of hydrogel crosslinks via hydrolysis or enzymatic activity determines the rate of drug liberation. The degradation rate can be modulated by adjusting the chemical composition and the degree of crosslinking within the hydrogel, thereby providing a predictable release profile over days, weeks, or even months (Yacoub et al., 2022).

## 8 Preclinical and translational outcomes of hydrogel-based SMTE

A growing body of work has evaluated hydrogel−based and related SMTE strategies in *in vivo* animal models of VML and large skeletal muscle defects in human trials (Sicari et al., 2014; Garg et al., 2015; Grasman et al., 2015b; Sicherer et al., 2020; Eugenis et al., 2021). To date, acellular and simple cell−laden natural hydrogels (fibrin, collagen, dECM) have produced the most robust and reproducible *in vivo* functional gains, with drug−loaded variants showing promising incremental improvements. Acellular ECM bioscaffolds and collagen–GAG matrices in rodent VML models consistently improve muscle fiber regeneration, reduce fibrosis, and restore a substantial fraction of torque or tetanic force versus untreated defects (Zhu et al., 2023a). Porcine urinary bladder ECM scaffold implantation was associated with perivascular stem cell mobilization and accumulation within the site of injury, and *de novo* formation of skeletal muscle cells in both a preclinical rodent model and five male patients (Sicari et al., 2014). The ECM-mediated constructive remodeling was associated with stimulus-responsive skeletal muscle in rodents and functional improvement in three of the five human patients. Further, in a 13-patient clinical trial (NCT01292876) on VML, acellular bioscaffolds composed of mammalian extracellular matrix (ECM) combined with physical therapy improved strength and function and promoted site-appropriate remodeling of VML defects (Dziki et al., 2016). By 6 months after ECM implantation, patients showed an average improvement of 37.3% (P<0.05) in strength and 27.1% improvement in range-of-motion tasks (P<0.05) (Dziki et al., 2016).

Cell−based strategies using satellite cells, muscle−derived progenitors, or mesenchymal stromal cells delivered within natural or hybrid hydrogels improve vascularization and neuromuscular junction reformation in animal models and can augment torque production and endurance relative to scaffold−only controls (Eugenis et al., 2021; Gahlawat et al., 2024). However, long−term engraftment and contribution of transplanted cells to the mature myofiber pool remain limited, and host immune responses, matrix remodeling, and mechanical mismatch frequently constrain durable recovery (Sicherer et al., 2020). Emerging nanoparticle− and drug−loaded hydrogels further modulate inflammation and fibrosis, for example, by delivering IGF−1 or anti−fibrotic small molecules, and have yielded additional gains in contractile force and reduced collagen deposition in small−animal VML models (Harris et al., 2021; Lee et al., 2026).

Overall, current SMTE approaches demonstrate meaningful but incomplete functional rescue in preclinical models. Key obstacles include insufficient and disorganized vascular and neural integration, persistent fibrotic encapsulation, limited scalability to large defects, and gaps between small−animal outcomes and large−animal or human physiology. The most promising paths toward clinical translation appear to involve combinatorial strategies integrating (i) biomimetic scaffolds that restore a pro−regenerative niche, (ii) myogenic or stem/progenitor cells, and (iii) precisely timed delivery of growth factors, cytokines, or small molecules to balance myogenesis and fibrosis. Larger−animal studies, standardized functional testing, and rigorously designed early−phase clinical trials will be required to define which design principles most effectively translate to durable recovery in humans.

## 9 Limitations and challenges

Despite rapid advances and growing preclinical evidence, widespread clinical adoption of drug- and nanoparticle-loaded hydrogels for skeletal muscle regeneration remains constrained by key challenges in material design, manufacturing, immunological compatibility, and model translation. A central concern is the long-term biocompatibility of embedded nanoparticles, particularly non-biodegradable or slowly degrading materials such as certain metallic and silica nanoparticles, which may accumulate in tissues or chronically activate immune responses after implantation. Achieving hydrogel matrices that are simultaneously immunologically inert and mechanically compatible with dynamic skeletal muscle is inherently material-specific and requires careful tuning of biochemical composition and mechanical properties.

Scalability and reproducibility also pose substantial barriers, as many hydrogel–nanoparticle systems are fabricated under tightly controlled laboratory conditions, often using complex, multi-step syntheses or 3D bioprinting workflows that are difficult to translate into GMP-compliant, batch-consistent manufacturing. Combination products that integrate biomaterials with nanomaterials must navigate evolving regulatory frameworks for safety, quality control, and approval, further slowing bench-to-bedside translation. At the materials level, hydrogel mesh size and pore architecture critically regulate diffusion of nutrients, oxygen, growth factors, and ECM components, and typical mesh sizes (tens to hundreds of Å) can restrict transport in large or long-term constructs, forcing trade-offs between diffusivity and mechanical integrity. Bulk GelMA hydrogels with pore sizes of roughly 5–10 μm limit nutrient transport, cell spreading, and migration, whereas more porous GelMA scaffolds with larger, interconnected pores better support oxygen and nutrient diffusion but can weaken structural strength. Synthetic polymers such as PEG often lack intrinsic cell-adhesion motifs and resist protein adsorption. In contrast, biopolymers like collagen and fibrin provide integrin-binding ligands yet introduce batch variability, illustrating another design trade-off.

Additional issues include cytotoxicity associated with certain gelation or crosslinking methods (e.g., UV exposure, radical initiators, elevated temperature), spatial heterogeneity in cell distribution, and challenges in achieving uniform encapsulation across large constructs. Incorporating bioactive additives such as cellulose nanocrystals, graphene oxide, antioxidant enzymes, or glycosaminoglycan mimetics can enhance bioactivity and oxidative protection but also complicate formulation, quality control, and regulatory assessment. Post-implantation, hydrogels remain vulnerable to adverse immune reactions, fibrotic encapsulation, or incomplete degradation, each of which can impair tissue integration and functional recovery. An incomplete understanding of how specific matrix chemistries interface with cell signaling pathways limits the predictability of *in vivo* outcomes.

On the biological side, most preclinical studies still rely on immortalized cell lines, such as C2C12 myoblasts, which do not fully recapitulate the complexity of human muscle regeneration. However, newer work using primary human skeletal muscle cells or iPSC-derived myogenic cells offers greater translational relevance at the cost of greater technical demand. Three-dimensional culture systems introduce additional complexity, including more difficult medium exchange within cell aggregates or spheroids, increased risk of contamination, and challenges in imaging and biochemical analysis due to increased thickness, opacity, and heterogeneous diffusion of reagents. Quantifying cell number and phenotype within dense 3D constructs is non-trivial because cells are entrapped and often require harsh dissociation, prompting reliance on surrogate readouts, such as DNA or protein content, which can be biased by limited reagent penetration and signal attenuation. Although new culture vessels, automation, and standardized 3D assay protocols are being developed to address these limitations, they are not yet widely adopted and add cost and operational complexity.

## 10 Conclusions and future directions

Hydrogel technologies are rapidly progressing toward systems that more closely recapitulate the structural, mechanical, and biochemical complexity of native skeletal muscle, positioning drug- and nanoparticle-loaded hydrogels as powerful platforms for localized, sustained, and multimodal therapy. Emerging “smart” and responsive hydrogels are being engineered to sense local cues such as pH, ROS, enzyme activity, or mechanical strain and to modulate drug release or scaffold degradation, accordingly, thereby improving spatiotemporal control over inflammation resolution, angiogenesis, myogenesis, and tissue integration. In parallel, multifunctional nanoparticles are being designed to combine therapeutic delivery with imaging or electrical modulation, enabling real-time monitoring of tissue repair while simultaneously providing biochemical and biophysical cues.

The integration of stem cells and patient-specific iPSCs with hydrogel–nanoparticle platforms opens avenues for personalized regenerative therapies, particularly when combined with advanced 3D bioprinting strategies that allow precise spatial patterning of cells, vasculature-mimicking channels, and anisotropic architectures that better reflect native muscle organization. At the systems level, advances in AI-driven automation, robotics, and microfluidic culture systems are streamlining 3D tissue model generation, improving control over nutrient perfusion and biochemical gradients, and enhancing the physiological fidelity of *in vitro* models for drug screening and disease modelling. Regulatory trends, including the FDA’s recent support for organoid and organ-on-a-chip approaches as partial alternatives to animal testing, are likely to accelerate the adoption of such engineered tissues for preclinical safety and efficacy evaluations.

Building on these advances, the integration of organoid−based or organoid−like three−dimensional muscle constructs within hydrogel systems has emerged as an important direction in skeletal muscle regenerative engineering (Iberite et al., 2022; Auletta et al., 2025; Yang et al., 2025). These engineered muscle organoids, often derived from iPSCs or primary myogenic cells, can self−organize into aligned, multinucleated myofibers embedded in a supportive matrix and, when combined with vascular or neural components, more closely recapitulate the architectural complexity of native muscle (Messina et al., 2005; van der Wal et al., 2023). Organoid−integrated hydrogels offer several potential advantages for VML repair, including improved structural fidelity, enhanced engraftment stability within large defects, and a permissive niche for host vascular and neural ingrowth that may accelerate revascularization and reinnervation (Gholobova et al., 2020; Nwokoye and Abilez, 2024). At the same time, significant challenges remain: most muscle organoids are still limited in size and contractile output, batch−to−batch variability and incomplete maturation complicate standardization, and immunological compatibility, long−term survival, and functional integration after implantation remain unresolved (Liu et al., 2023; van der Wal et al., 2023). Future work will need to couple organoid technologies with immunomodulatory, pro−angiogenic hydrogels and advanced biofabrication (e.g., bioprinting of pre−vascularized or neuromuscular units) while developing scalable, Good Manufacturing Practice (GMP)−compatible manufacturing and rigorous functional benchmarking in large−animal VML models (Volpi et al., 2022).

Looking forward, progress in multimodal delivery systems capable of co-delivering small molecules, growth factors, cytokines, nucleic acids, and extracellular vesicles, along with bioinspired, stimuli-responsive hydrogels, is expected to drive the next generation of muscle regeneration therapies. However, translation to the clinic remains constrained by several key challenges, including scalable manufacturing of complex constructs, reliable vascular and neural integration within large VML defects, and demonstration of long−term functional durability (sustained force generation and resistance to fibrosis) in clinically relevant models. Realizing clinical impact will require robust large-animal studies, scalable and standardized manufacturing pipelines, long-term biocompatibility and biodistribution assessments (particularly for nanomaterials), and alignment with evolving regulatory and quality frameworks. As interdisciplinary collaborations among materials scientists, bioengineers, clinicians, and regulatory experts deepen, drug-loaded and nanoparticle-reinforced hydrogels are poised to transition from experimental platforms to clinically relevant, customizable treatments for volumetric muscle loss and other skeletal muscle injuries and degenerative disorders.
